# Targeting the CRYAB/β‐Catenin Axis with an MMP‐9‐Responsive Hydrogel Disrupts CTC Clusters to Suppress Progression of Hepatocellular Carcinoma after Radiofrequency Ablation

**DOI:** 10.1002/advs.77578

**Published:** 2026-09-16

**Authors:** Hui Chen, Danni Yang, Songying Pi, Yuhan Liu, Hongbin Huang, Feile Ye, Yuhong Lin, Zhongzhen Su, Yongquan Huang

**Affiliations:** ^1^ Department of Ultrasound The Fifth Affiliated Hospital of Sun Yat‐Sen University Zhuhai Guangdong Province China; ^2^ Guangdong Provincial Engineering Research Center of Molecular Imaging Guangdong‐Hong Kong‐Macao University Joint Laboratory of Interventional Medicine the Fifth Affiliated Hospital Sun Yat‐sen University Zhuhai China

**Keywords:** β‐catenin, circulating tumor cell, CRYAB, hepatocellular carcinoma, hydrogel, radiofrequency ablation

## Abstract

Incomplete radiofrequency ablation (iRFA) represents a major clinical challenge in hepatocellular carcinoma (HCC) management, as residual tumors acquire enhanced metastatic potential through undefined mechanisms. Herein, we report that sublethal thermal stress upregulates the heat‐responsive molecular chaperone CRYAB, which stabilizes β‐catenin protein by inhibiting its ubiquitin‐mediated proteasomal degradation. The CRYAB/β‐catenin axis drives epithelial‐mesenchymal transition (EMT) in residual HCC cells and simultaneously polarizes tumor‐associated neutrophils (TANs) toward the pro‐tumorigenic N2 phenotype. This dual regulation pre‐programs a pro‐metastatic microenvironment in situ, promotes the formation of heterotypic circulating tumor cell (CTC) clusters, and ultimately accelerates tumor recurrence and metastasis post‐iRFA. To suppress this pathological cascade, we engineered a matrix metalloproteinase‐9 (MMP‐9)‐responsive thermosensitive hydrogel system (XB@Gel) that enables localized and sustained co‐delivery of the β‐catenin inhibitor XAV‐939 and the PD‐L1 inhibitor BMS‐202. XB@Gel effectively reverses EMT, reprograms the immunosuppressive microenvironment, reduces CTC clusters, and robustly inhibits HCC recurrence and metastasis after iRFA. Collectively, our findings identify the CRYAB/β‐catenin axis as a key transducer of thermal stress into a pro‐metastatic signaling cascade in residual HCC and establish XB@Gel as a promising multi‐dimensional therapeutic strategy to combat post‐iRFA progression.

## Graphical abstract

The graphical abstract illustrates that sublethal thermal stress after iRFA upregulates the chaperone CRYAB in residual HCC cells, which stabilizes β‐catenin and drives a dual pathological program, including EMT and N2‐TAN polarization, thereby leading to heterotypic CTC cluster formation. The MMP‐9‐responsive hydrogel (XB@Gel) delivers XAV‐939 and BMS‐202 directly to the residual tumor, blocking this axis and reprogramming the immune microenvironment, ultimately inhibiting local recurrence and metastasis.

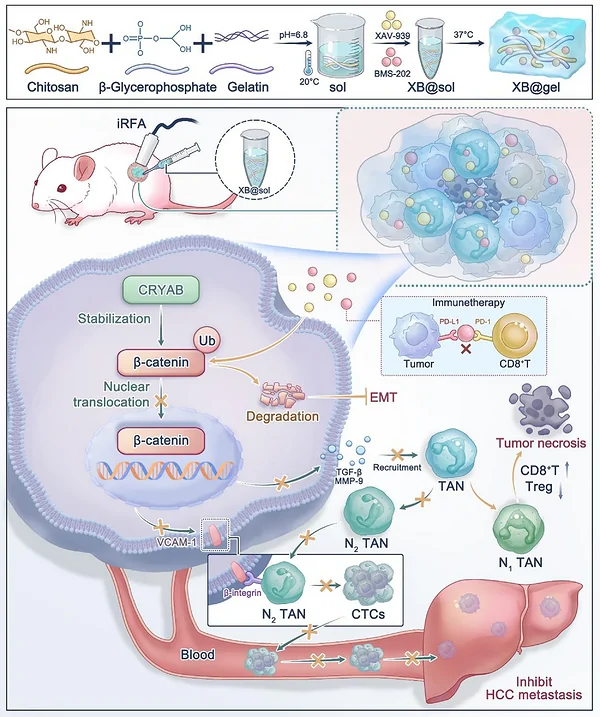

## 1 Introduction

Hepatocellular carcinoma (HCC) is one of the leading causes of cancer‐related mortality worldwide [1]. Due to its insidious onset and rapid progression, the majority of HCC patients are diagnosed at stages where curative surgical resection is no longer feasible [2]. Radiofrequency ablation (RFA), owing to its minimally invasive nature, favorable safety, short hospital stay, and reliable therapeutic efficacy, has become a first‐line locoregional therapy for patients with early‐stage or unresectable HCC [3, 4]. However, restricted by clinical factors such as the heat sink effect, tumor heterogeneity, and microvascular invasion, the incidence of incomplete RFA (iRFA) ranges from 30% to 50% [5]. The recurrence and metastasis of residual tumor cells following iRFA have emerged as the central bottleneck limiting long‐term patient survival [6].

Accumulating evidence suggests iRFA‐induced residual tumors are not dormant [7]. Sublethal thermal stress induced by iRFA triggers sustained biological signaling, driving malignant progression of residual lesions [8, 9]. Our previous work demonstrates that sublethal thermal stress directly induces epithelial‐mesenchymal transition (EMT) in residual HCC cells, facilitating metastatic potential [10]. We also reported that thermal injury of residual tumor cells recruits and polarizes tumor‐associated neutrophils (TANs) toward the pro‐tumor N2 phenotype [11, 12]. Recent studies have shown that N2‐polarized TANs secrete matrix metalloproteinase‐9 (MMP‐9) and transforming growth factor‐β (TGF‐β), which promote EMT of cancer cells [13]. These findings suggest that the malignant transformation of residual cancer cells and the immune remodeling of the tumor microenvironment (TME) post‐iRFA are tightly and coordinately regulated. Critically, tumor cells and TANs can interact via cell‐cell adhesion and paracrine signaling to form heterogeneous circulating tumor cell (CTC) clusters [14, 15]. Moreover, both mesenchymal‐like tumor cells and N2‐polarized TANs exhibit a higher propensity for such cluster formation [16]. Compared with single CTC, heterogeneous CTC clusters possess enhanced circulatory survival and immune evasion, acting as efficient metastatic “seeds” and key initiators of dissemination [17]. Thus, elucidating the cooperative regulatory network between residual HCC and TANs and identifying the molecular hubs is critical to mitigating post‐RFA tumor invasion and metastasis.

RFA inactivates tumors through local hyperthermia, yet sublethal thermal stress during iRFA induces stress‐related alterations in residual tumors [18]. As a member of the small heat shock protein family, αB‐crystallin (CRYAB) functions as a molecular chaperone that serves as an important protective molecule against external injuries such as thermal stress [19]. It promotes tumor proliferation, metastasis, and drug resistance by maintaining the stability of pro‐tumorigenic proteins [20]. Existing studies have demonstrated that CRYAB is upregulated in tumor cells and is closely associated with poor prognosis in patients [21]. However, no study has yet shown a link between CRYAB and iRFA‐mediated alterations in the immune TME or residual tumor metastasis, and how CRYAB regulates these processes remains unclear.

Among molecules involved in tumor progression, β‐catenin exerts dual functions in regulating EMT and tumor immune evasion, representing a key target with great clinical translational potential [22, 23]. As a central component of the canonical Wnt signaling pathway, aberrant activation of β‐catenin typically arises from pathway dysregulation or genetic mutations and can induce EMT by suppressing E‐cadherin expression while concurrently participating in tumor immune evasion and resistance to immunotherapy [24, 25]. Although our previous work confirmed that sublethal thermal stress downregulates E‐cadherin expression [10], critical questions remain unresolved, including whether thermal stress mediates EMT in residual HCC cells through β‐catenin activation. In addition, the specific mode of β‐catenin activation and its role in remodeling the immune TME have yet to be determined post‐iRFA.

Immunotherapy serves as an important systemic therapeutic modality for HCC after iRFA, yet it still faces several limitations in clinical practice: systemic drug therapy suffers from poor targeting and substantial toxicity; immune checkpoint inhibitor monotherapy yields limited efficacy due to the immunosuppressive microenvironment; and conventional therapies are unable to achieve precise, long‐acting drug delivery [26, 27]. More critically, existing strategies lack multi‐dimensional interventional approaches capable of simultaneously blocking tumor cell malignant transformation, immunosuppression, and metastatic initiation. In this study, we identify the CRYAB/β‐catenin axis as a critical driver of iRFA‐induced residual HCC progression and engineer an MMP‐9‐responsive hydrogel for local co‐delivery of a β‐catenin inhibitor and a PD‐L1 immune checkpoint inhibitor (Scheme 1). This combinatorial platform enables precise residual tumor targeting and effectively blocks HCC recurrence and metastasis after iRFA.

## 2 Materials and Methods

### 2.1 Reagents

Chitosan (Mw 100–300 kDa, degree of deacetylation ≥95%, purity ≥99%) was purchased from Macklin (China; C6340). Gelatin (Type A, from porcine skin, gel strength ∼300 g Bloom) was purchased from Vetec (Sigma‐Aldrich, USA; V900863‐100G). Acetic acid (>99.5% purity) was purchased from TCI (Tokyo Chemical Industry, Tokyo, Japan; A2035). β‐Glycerophosphate (β‐GP, Mw 306.11, analytical reagent [AR] grade, purity >99%) was purchased from Dalian Meilun Biotechnology Co., Ltd. (China; O1016E). Sodium bicarbonate (NaHCO_3_, MW 84.01, analytical reagent [AR] grade, purity 99.8%) was purchased from Yuanye Biotechnology Co., Ltd. (China; A09GS14851). XAV‐939 (purity ≥99.5%) was obtained from MedChemExpress (USA, HY‐15147). BMS‐202 (purity ≥99%) was obtained from Selleck Chemicals (USA, S7912). BML‐284 (purity ≥98%) was obtained from TargetMol (USA, 2095432‐75‐8). MG132 (purity ≥98%) was obtained from InvivoGen (USA, tlrl‐mg132‐2).

### 2.2 Cell Lines and Animals

Human hepatocellular carcinoma cell lines Hep3B and MHCC97H, murine hepatocellular carcinoma cell lines H22 and Hepa1‐6, as well as H22 cell lines stably expressing luciferase or GFP, were obtained from the Cell Bank of the Chinese Academy of Sciences (Shanghai, China). All cells were cultured in the recommended media and maintained at 37°C in a humidified atmosphere containing 5% CO_2_.

Female BALB/c mice (3–4 weeks old) were purchased from Guangdong Yaokang Biotechnology Co., Ltd. and maintained under specific pathogen‐free (SPF) conditions until 8 weeks of age for subsequent experiments. All animal experimental protocols were approved by the Experimental Animal Center of the Fifth Affiliated Hospital of Sun Yat‐sen University (Approval No. 2024060401) and performed in accordance with relevant guidelines and regulations.

### 2.3 Cell Transfection

Small interfering RNAs (siRNAs) were synthesized by Guangzhou RiboBio Co., Ltd., including a negative control (si‐NC) and three CRYAB‐targeting sequences: CRYAB‐a (GGATCCCAGCTGATGTAGA), CRYAB‐b (CTGTCAACCTGGATGTGAA), and CRYAB‐c (CCATCACCCGTGAAGAGAA). siRNAs were dissolved in RNase‐free water to prepare a 20 µmol/L stock solution, aliquoted, and stored at −20°C. Transfections were performed using Lipofectamine 2000 reagent according to the manufacturer's instructions.

### 2.4 Plasmid Construction

Plasmid construction was performed following the protocols provided by the reagent supplier. The pLV3‐CMV and CMV‐MCS lentiviral systems were used to generate expression constructs. The full‐length Cryab coding sequence (NM_009964) with a His tag was amplified by PCR and inserted into the pLV3‐CMV‐Cryab (mouse) ‐3×FLAG‐Fluc‐Puro vector.

### 2.5 Lentivirus‐Mediated Stable Overexpression

The OE‐Cryab plasmid or empty vector was co‐transfected into HEK293T cells with psPAX2 and pMD2.G at a ratio of 4:3:1. Viral supernatants were collected at 48 h and 72 h post‐transfection, filtered through a 0.45 µm membrane, and used to infect H22 cells. After 48–72 h of infection, puromycin (5 µg/mL) was added for selection for approximately 10 days until all cells in the non‐infected control group were eliminated. Surviving cells were expanded to establish stable Cryab‐overexpressing cell lines.

### 2.6 Gene Knockout

CRYAB‐knockout H22 cells were generated using the CRISPR/Cas9 system. Specific sgRNAs targeting Cryab were designed and cloned into the pLV3‐U6 vector. Cells were transfected and selected with puromycin (5 µg/mL) to obtain positive clones. Single‐cell clones were expanded and validated by Sanger sequencing and Western blotting to confirm knockout efficiency.

### 2.7 Quantitative Real‐Time PCR (Q‐PCR)

Total RNA was extracted using FreeZol reagent (Vazyme, R711) according to the manufacturer's instructions. Reverse transcription was performed using HiScript III RT SuperMix (Vazyme, R323) to generate cDNA. Quantitative PCR was conducted using SYBR Green Master Mix (Yeasen, 11202ES) on a real‐time PCR system. Primer sequences are provided in the Supplementary Materials.

### 2.8 Transcriptome Sequencing

Hep3B cells were incubated at 37°C or 44°C for 24 h and then harvested for analysis. Total RNA was extracted using TRIzol reagent (Invitrogen) according to the manufacturer's instructions. cDNA library construction and RNA sequencing were performed on an Illumina platform by Guangzhou RiboBio Co., Ltd. Differentially expressed genes (DEGs) were identified using a threshold of false discovery rate (FDR) < 0.05 and |log_2_ fold change (|log_2_FC|) > 1.

### 2.9 Bioinformatics Analysis

The prognostic significance of CRYAB in hepatocellular carcinoma was evaluated using the Kaplan–Meier Plotter database (http://kmplot.com/analysis/). The protein–protein interaction (PPI) network of CRYAB was constructed using the STRING database (version 12.0, https://string‐db.org/).

### 2.10 Preparation of Tumor‐Conditioned Medium (TCM)

To obtain TCM, HCC cells were seeded in 6‐well plates and cultured for 24 h. The medium was then replaced with serum‐free medium, and a sublethal heat stress model was established according to our previous studies [10, 28]. Briefly, culture plates were sealed with Parafilm and immersed in a preheated water bath at 37°C or 44°C for 15 min. After treatment, cells were returned to a 37°C incubator with 5% CO_2_ and cultured for an additional 48 h. The supernatant was collected and centrifuged to remove cellular debris, followed by filtration through a 0.22 µm membrane. The conditioned medium was aliquoted and stored at −20°C for further use.

### 2.11 Cell Migration and Invasion Assays

Cell migration and invasion abilities were assessed using Transwell chambers with 8 µm pore size (Corning) following siCRYAB treatment.

Cells were resuspended in serum‐free medium at a density of 2 × 10^5^/mL. For the migration assay, 200 µL of cell suspension was added to the upper chamber, while 600 µL of complete DMEM containing 10% fetal bovine serum (FBS) was added to the lower chamber. After 24 h of incubation, non‐migrated cells were removed with a cotton swab. Cells were fixed with 4% paraformaldehyde for 15 min and stained with 1% crystal violet for 30 min. Images were acquired under a microscope, and cells were counted in multiple random fields.

For the invasion assay, the upper chamber was pre‐coated with Matrigel (BD, 356234) diluted according to the manufacturer's instructions and polymerized at 37°C. The remaining procedures were identical to the migration assay, with incubation extended to 24–48 h. The number of invaded cells was quantified after fixation and staining.

### 2.12 Western Blot Analysis

Cells were lysed according to experimental groups, and total protein was extracted. Protein samples were denatured and separated by 10% SDS‐PAGE, followed by transfer onto PVDF membranes using a Mini Trans‐Blot system. Membranes were blocked and incubated overnight at 4°C with primary antibodies against CRYAB (CST, D6S9E), VCAM‑1 (Abcam, ab134047), Vimentin (CST, 5741), E‑cadherin (CST, 14472), N‑cadherin (NOVUS, NB100‐92447), β‑catenin (CST, D10A8). After washing, membranes were incubated with HRP‐conjugated secondary antibodies (Zhongshan Jinqiao, China) at room temperature. Protein bands were visualized using enhanced chemiluminescence (ECL) detection system.

### 2.13 Co‐Immunoprecipitation (Co‐IP)

HCC cells treated with heat stress (44°C) or control conditions (37°C) were harvested and lysed on ice. Protein concentration was determined, and 1000 µg of total protein lysate was incubated with anti‐CRYAB antibody (CST, D6S9E; 1:50 dilution) at 4°C overnight to form antigen–antibody complexes. Protein A magnetic beads (30 µL) were washed with PBS and blocked with 1% BSA at 4°C for 1 h. The antibody–protein complex was then incubated with pretreated beads at room temperature for 1 h with rotation. Beads were collected using a magnetic rack, and the supernatant was discarded.

Beads were washed three times with 200 µL RIPA buffer and resuspended in 1× loading buffer, followed by denaturation at 100°C for 15 min. The eluted proteins were subsequently analyzed by Western blotting.

### 2.14 Cytoplasmic and Nuclear Protein Extraction

Cytoplasmic and nuclear fractionation was performed using a Nuclear and Cytoplasmic Protein Extraction Kit (Beyotime, P0027) according to the manufacturer's instructions. HCC cells collected before and after heat stress treatment were used for analysis. Cell pellets were resuspended in 200 µL cytoplasmic protein extraction reagent A and vortexed vigorously, followed by incubation on ice. Then, 10 µL cytoplasmic protein extraction reagent B was added, followed by vortexing, ice incubation, and centrifugation. The supernatant was collected as the cytoplasmic fraction. The remaining pellet was resuspended in 50 µL nuclear protein extraction reagent and vortexed intermittently (10 s every 5 min) during ice incubation. After centrifugation, the supernatant was collected as the nuclear fraction. Equal amounts of cytoplasmic and nuclear proteins were subjected to Western blot analysis. Histone H3 (CST, 9715) was used as a nuclear loading control.

### 2.15 Immunofluorescence Assay (CRYAB/β‐Catenin Co‐Localization)

HCC cells were seeded on glass‐bottom confocal dishes and cultured for 24 h, followed by exposure to heat stress at 44°C (control group: 37°C). After 24 h, cells were washed and fixed with 4% paraformaldehyde at room temperature. Cells were permeabilized with PBS containing 0.5% Triton X‐100 for 30 min, followed by blocking with 3% BSA for 2 h at room temperature. Cells were then incubated overnight at 4°C with primary antibodies against CRYAB (mouse, Abcam, 6D11; 1:200) and β‐catenin (rabbit, CST, D10A8; 1:200). After washing, cells were incubated with Alexa Fluor 488‐conjugated goat anti‐rabbit IgG (Invitrogen, A11013; 1:200) and Alexa Fluor 647‐conjugated goat anti‐mouse IgG (Abcam, Ab150115; 1:200) at room temperature in the dark for 1 h. Nuclei were counterstained with DAPI‐containing antifade mounting medium. Images were acquired using a laser scanning confocal microscope to assess intracellular localization and co‐localization of CRYAB and β‐catenin.

### 2.16 ELISA Assay

Enzyme‐linked immunosorbent assay (ELISA) was performed using commercial kits (MMP‐9: EK2M09; G‐CSF: EK269; TGF‐β: EK981; LIANKE Biotechnology) according to the manufacturer's instructions. Cell culture supernatants or mouse serum samples were collected. For serum collection, blood was obtained via retro‐orbital sampling and centrifuged at 4°C, 3000 × *g* for 15 min to obtain serum. Samples were appropriately diluted before analysis. After plate blocking, 100 µL of standards or samples were added per well and incubated at 37°C for 1–2 h, followed by washing three times. Biotinylated antibodies and enzyme conjugates were sequentially added (100 µL per well) and incubated at 37°C for 1 h and 30 min, respectively, with washing steps in between. TMB substrate was added for color development (37°C, 10–30 min in the dark), and the reaction was stopped before measuring absorbance at 450 nm. Target protein concentrations were calculated based on standard curves.

### 2.17 Neutrophil Isolation

Neutrophils were isolated from mouse bone marrow using a Percoll density gradient centrifugation method according to our previous studies [11]. Briefly, mice were euthanized, and femurs and tibias were collected. Bone marrow cells were flushed with PBS and resuspended. Percoll solutions (78%, 65%, and 55%) were sequentially layered (2 mL each), and the cell suspension (2 mL) was carefully added on top. After centrifugation, cells located at the interface between 78% and 65% Percoll were collected as neutrophils and used for further experiments.

### 2.18 Neutrophil Migration Assay

Neutrophil chemotactic migration was evaluated using Transwell chambers with 3 µm pore size (Corning). Freshly isolated neutrophils were resuspended in serum‐free RPMI‐1640 medium containing 1% PBS and adjusted to a density of 1 × 10^6^ cells/mL. A total of 100 µL of cell suspension was added to the upper chamber, while TCM from different groups (Control, Heat, Heat + siCryab‐a, and Heat + siCryab‐b) was added to the lower chamber. Care was taken to avoid air bubbles. After incubation for 6 h, cells were fixed with 4% paraformaldehyde for 15 min and stained with 1% crystal violet for 30 min. Migrated cells were imaged under a light microscope and quantified as the percentage of migrated cells.

### 2.19 Immunofluorescence Analysis of Neutrophil Polarization Morphology

To assess cytoskeletal remodeling and polarization status, neutrophils were seeded onto poly‐L‐lysine‐coated coverslips (14 mm diameter, LABSELECT) placed in 24‐well plates. Coverslips were pretreated with 1 mg/mL poly‐L‐lysine (prepared in PBS) at 37°C for 1 h. Neutrophil suspensions were carefully added onto the center of the coverslips and allowed to adhere, followed by incubation with TCM from different groups at 37°C for 24 h. Cells were then washed twice with PBS and fixed with 4% paraformaldehyde for 15 min. Permeabilization was performed using PBS containing 3% BSA for 15 min. F‐actin was stained with phalloidin‐FITC (ABclonal), and nuclei were counterstained with DAPI. Fluorescence signals were acquired using a confocal laser scanning microscope under 488 nm excitation.

### 2.20 Neutrophil Functional Assays

Neutrophil polarization assay: To evaluate the causal role of TGF‐β and MMP‐9 in CRYAB‐mediated TAN polarization, neutrophils were co‐cultured with TCM derived from heat‐stressed or CRYABOE HCC cells, with or without the TGF‐β receptor inhibitor SB‐431542 (10 µm) or the MMP‐9 inhibitor SB‐3CT (10 µm). After 24 h of incubation, cells were harvested and stained with PerCP‐CD45 (103130), AF700‐CD11b (557960), FITC‐Ly6G (127606), PE‐iNOS (696805), and BV421‐CD206 (141717). TAN polarization was then analyzed by flow cytometry, and the percentages of CD206^+^ and iNOS^+^ cells within the Ly6G^+^ neutrophil population were quantified.

Neutrophil–tumor cell adhesion assay: To evaluate the adhesion capacity between neutrophils and Hepa1‐6 cells, neutrophils and tumor cells were labeled with DiO and DiD fluorescent dyes, respectively, for 20 min. The two cell types were then co‐cultured at a 10:1 ratio (neutrophils: tumor cells) at 37°C for 2 h. After incubation, non‐adherent cells were removed by washing three times with PBS, and nuclei were counterstained with Hoechst. Cell–cell adhesion was visualized and recorded using a confocal laser scanning microscope.

### 2.21 Preparation of XB@Gel Hydrogel

Chitosan (1 g) was dissolved in 50 mL of deionized water containing 0.1 m acetic acid at room temperature. After a homogeneous chitosan solution was obtained, gelatin (0.25 g) was slowly added under magnetic stirring at 400 rpm, followed by continuous stirring for 4 h until a homogeneous solution was obtained. The resulting chitosan/gelatin solution was then sterilized by autoclaving. A 44.4% (w/v) β‐glycerophosphate (β‐GP) solution was prepared separately in deionized water and supplemented with 0.1 M NaHCO3 under an ice bath. At 4°C, the β‐GP solution was slowly added dropwise to the chitosan/gelatin solution under continuous stirring until the pH of the resulting mixture reached 6.8. The resulting thermosensitive hydrogel (designated as Gel) was stored at 4°C as a drug delivery carrier [29]. For drug‐loaded hydrogel preparation, XAV‐939 and BMS‐202 were added to Gel and thoroughly mixed at 4°C to obtain XB@Gel. To systematically evaluate the effect of gelatin incorporation on formulation performance, we prepared hydrogels with a fixed chitosan concentration and varying gelatin concentrations (0%, 0.25%, 0.5%, 1.0%, and 2.0%) and assessed mechanical properties and drug release kinetics.

### 2.22 Physicochemical Characterization of XB@Gel

Gelation time was determined using the vial inversion method [30]. Briefly, the solution was placed in a 2 mL vial and inverted periodically until no flow was observed. The gelation time was recorded as the time point at which flow ceased. Each measurement was performed in triplicate. Injectability and gel formation were evaluated at 37°C. Freeze‐dried hydrogels were analyzed for microstructure using scanning electron microscopy (Phenom Pure). Rheological properties were measured using a strain‐controlled rheometer (Waters DHR‐2). Samples (1 mL) were placed between parallel plates (40 mm diameter, 1 mm gap) and analyzed at 37°C under a fixed strain of 1%. Frequency sweeps were performed over the range of 0.1–100 Hz to evaluate viscoelastic behavior. For the X@Gel and XB@Gel formulations, drug encapsulation efficiency was determined using a hydrogel dry‐weight method. Briefly, drug‐free hydrogel, X@Gel containing XAV‐939 (1.05 mg/mL), and XB@Gel containing XAV‐939 (1.05 mg/mL) and BMS‐202 (10 mg/mL) were prepared and allowed to gel at 37°C in a water bath. The formed hydrogels were washed three times with PBS to remove unencapsulated drugs, followed by freezing at −80°C for 4 h and lyophilization for 12 h. The dry weight of each resulting hydrogel powder was recorded. Drug encapsulation efficiency (EE) was calculated using the following equation: EE (%) = [(W_loaded_ − W_blank_) / W_initial drug_] × 100%, where W_loaded_ represents the dry weight of the drug‐loaded hydrogel, W_blank_ represents the dry weight of the corresponding drug‐free hydrogel, and W_initial drug_ represents the initial mass of drug added.

### 2.23 In Vitro Experiment‐ on Drug‐ Release‐ and MMP‐9‐Responsive Assay

One milliliter of Gel was mixed with 1 mg FITC and incubated at 37°C to form a hydrogel system. To remove loosely bound FITC, the hydrogels were washed three times with PBS until no fluorescent signal was detected in the supernatant, confirming complete removal of unbound dye. Subsequently, 200 µL of preheated PBS or PBS containing 25 nm MMP‐9 was added onto the hydrogel surface. To verify MMP‐9‐dependent release, control groups were included: (1) PBS alone, (2) MMP‐9 + MMP‐9 inhibitor SB‐3CT (10 µm), and (3) 10% FBS in PBS. At predetermined time points, 10 µL of supernatant was collected and replaced with an equal volume of fresh PBS to maintain system stability. Absorbance at 490 nm was measured using a UV–vis spectrophotometer. Meanwhile, hydrogel height (h) and diameter (d) were recorded to calculate the volume by the formula: $V\;=\frac{\pi \times d^{2}\times h}{4}\;$. FITC concentrations were calculated based on a standard curve, and cumulative release profiles were determined by integrating concentration and hydrogel volume changes until complete release was achieved.

### 2.24 In Vivo Experiment‐ on Drug‐ Degradation‐ and Release‐

To evaluate in vivo degradation behavior, 100 µL of XB@Gel was subcutaneously injected into mice. Hydrogel remnants were harvested on days 1, 3, 7, and 14 post‐injection, followed by lyophilization and weighing to assess degradation kinetics.

To evaluate in vivo MMP‐9‐responsive drug release, ICG‐loaded hydrogel (ICG@Gel) was prepared by mixing ICG (1 mg/mL) into the gel precursor solution. Mice bearing subcutaneous sham‐treated or iRFA‐treated tumors received intratumoral injection of 50 µL ICG@Gel (n = 3). Fluorescence signals were monitored using an IVIS Spectrum imaging system at 0, 3, 7, and 14 days post‐injection. Fluorescence intensity was quantified using Living Image software.

### 2.25 Biocompatibility Evaluation of Hydrogel

Hemolysis assay was performed to evaluate the blood compatibility of the materials. Fresh anticoagulated blood (1 mL) was collected from BALB/c mice with heparin (10 U) and diluted with an equal volume of normal saline as the baseline sample. Four groups were established (*n* = 3): Gel group and XB@Gel group (samples washed and incubated in 8 mL normal saline, respectively), negative control (normal saline), and positive control (distilled water). After pre‐incubation at 37°C for 30 min, 0.2 mL of diluted blood was added to each group, followed by incubation for an additional 1 h. Samples were then centrifuged at 2000 g for 10 min, and the supernatant was collected for absorbance measurement at 545 nm. Hemolysis rate was calculated using the formula: Hemolysis (%) = (A − B)/(C − B) × 100%, where A, B, and C represent the absorbance of the experimental group, negative control, and positive control, respectively. A hemolysis rate below 5% was considered indicative of good biocompatibility.

### 2.26 Establishment of Mouse Tumor Models

H22 cells were subcutaneously injected into the dorsal flank of BALB/c mice to establish a tumor model. When tumor diameters reached 5–8 mm, an incomplete radiofrequency ablation (iRFA) model was established under anesthesia and ultrasound guidance by inserting the RFA electrode into the tumor center (located at the anterior one‐third of the long axis) and applying 5 W power for 20 s. The hepatic metastasis model was established via intrasplenic injection of 1 × 10^6^ Luc‐H22 cells, which entered the liver through the portal vein and formed metastatic lesions. For therapeutic evaluation, mice were randomly divided into six groups (n = 5): Con (iRFA + PBS), Gel (iRFA + Gel), XAV (iRFA + XAV‐939), X@Gel (iRFA + X@Gel), B@Gel (iRFA + B@Gel), and XB@Gel (iRFA + XB@Gel) groups. Immediately after iRFA, 50 µL of corresponding formulations were injected into the residual tumor region. The doses of XAV‐939 and BMS‐202 were 0.42 mg/kg and 4 mg/kg, respectively. Tumor volume was calculated using the formula: V = (width^2^ × length)/2. On day 8, tumors were harvested for flow cytometric analysis, and hepatic metastasis was monitored using an IVIS imaging system. On day 21, mice were sacrificed, and major organs were collected for H&E staining and serum biochemical analysis.

### 2.27 Flow Cytometry Analysis

All antibodies used for flow cytometry were purchased from BioLegend (USA). Tumor‐bearing mice were sacrificed on day 8 post‐treatment, and tumor tissues were collected to assess changes in the immune microenvironment. The spleens were harvested on day 14. Tumor and spleen tissues were digested with type IV collagenase to generate single‐cell suspensions, followed by filtration through a 40 µm mesh. Cells were processed using a Fixation/Permeabilization kit (BD) according to the manufacturer's instructions. Cells were then stained with a panel of fluorescent antibodies, including PerCP‐CD45 (103130), AF700‐CD11b (557960), FITC‐Ly6G (127606), PE‐iNOS (696805), BV421‐CD206 (141717), APC‐CD3 (100236), FITC‐CD4 (100406), PE/Cy7‐CD8a (100722), BV605‐CD25 (102035), PE‐Foxp3 (320008), PE‐β‑integrin (103705). Data were acquired using a CytoFLEX LX flow cytometer and analyzed with FlowJo software.

### 2.28 Immunohistochemistry (IHC)

Tumor tissues were fixed in 4% paraformaldehyde, embedded in paraffin, and sectioned at approximately 3.5 µm thickness. Sections were deparaffinized, rehydrated, and subjected to antigen retrieval. Sections were incubated overnight at 4°C with primary antibodies against CRYAB, VCAM‐1, Vimentin, E‐cadherin, N‐cadherin, and β‐catenin (the sources and catalog numbers are the same as described above), followed by incubation with HRP‐conjugated secondary antibodies (MaxVision) at room temperature for 2 h. Stained slides were scanned using a 3D Histech digital slide scanner. Image analysis was performed using CaseViewer and ImageJ software.

### 2.29 Isolation of Circulating Tumor Cells (CTCs)

CTCs were isolated from the peripheral blood of GFP‐H22 tumor‐bearing mice using a size‐based filtration method. Peripheral blood was collected from the retro‐orbital venous plexus and processed immediately. Blood was collected at a volume of 70 µL/g body weight per mouse and resuspended in 5 mL PBS. Samples were then passed through a negative‐pressure filtration device using a polycarbonate membrane with a pore size of 10 µm (Whatman). Tumor cells with larger diameters were retained on the membrane surface, while blood cells passed through the pores. After filtration, cells on the membrane were fixed with 4% paraformaldehyde for 15 min. CTCs were identified based on GFP fluorescence. Samples were either directly subjected to confocal microscopy or further processed by red blood cell lysis for quantitative analysis using flow cytometry.

To validate the filtration‐based CTC enumeration with an orthogonal approach, mouse peripheral blood samples from the control and iRFA groups were submitted to Shanghai Na'ao Biotechnology, where CTCs were isolated using the NextCTC third‐generation non‐antibody‐dependent nanomicrofluidic circulating tumor cell capture platform. Enriched CTCs were prepared on slides by the company, and we subsequently performed staining procedures. These samples were fixed and stained with an anti‐VCAM‐1 antibody (39036T), anti‐Ly6G antibody (1A8), and DAPI to identify VCAM‐1^+^ CTCs and CTC–TAN clusters.

### 2.30 Molecular Docking and Molecular Dynamics (MD) Simulation

Molecular docking between CRYAB (PDB: 2WJ7) and β‐catenin (PDB: 8Z0U) was performed using HDOCK, which employs a hybrid docking strategy and generated 100 binding poses. The model with the lowest docking score (ITScorePP) was selected for subsequent 100‐ns molecular dynamics (MD) simulation. MD simulation was conducted using GROMACS 2026.3 with the Amber99 force field and TIP4P water model. RMSD, RMSF, SASA, Rg, hydrogen bonds, and binding free energy (MMPBSA) were analyzed using GROMACS built‐in modules. The computational analysis was performed by Huasuan Technology Co., Ltd.

### 2.31 Statistical Analysis

All experiments were independently repeated at least three times, and data are presented as mean ± standard deviation (SD). Sample sizes (n) are indicated in each figure legend and represent biological replicates unless otherwise stated. For comparisons between two groups, a two‐sided Student's *t*‐test was applied after confirming normality (Shapiro–Wilk test) and homogeneity of variance (Levene's test); otherwise, the non‐parametric Mann–Whitney U test was used. For multiple‐group comparisons, one‐way analysis of variance (ANOVA) with Tukey's post‐hoc test was used for normally distributed data with equal variances; the Kruskal–Wallis test followed by Dunn's post‐hoc test was applied for non‐normally distributed data. A *p*‐value < 0.05 was considered statistically significant. All statistical analyses were performed using GraphPad Prism 9.0 software. The testing level (α) was set at 0.05. For all ANOVA analyses, the assumption of normality and homogeneity of variance was verified prior to analysis.

## 3 Results

### 3.1 CRYAB Is Elevated in Residual HCC Post‐iRFA and Drives EMT and Metastasis

**FIGURE 1:**
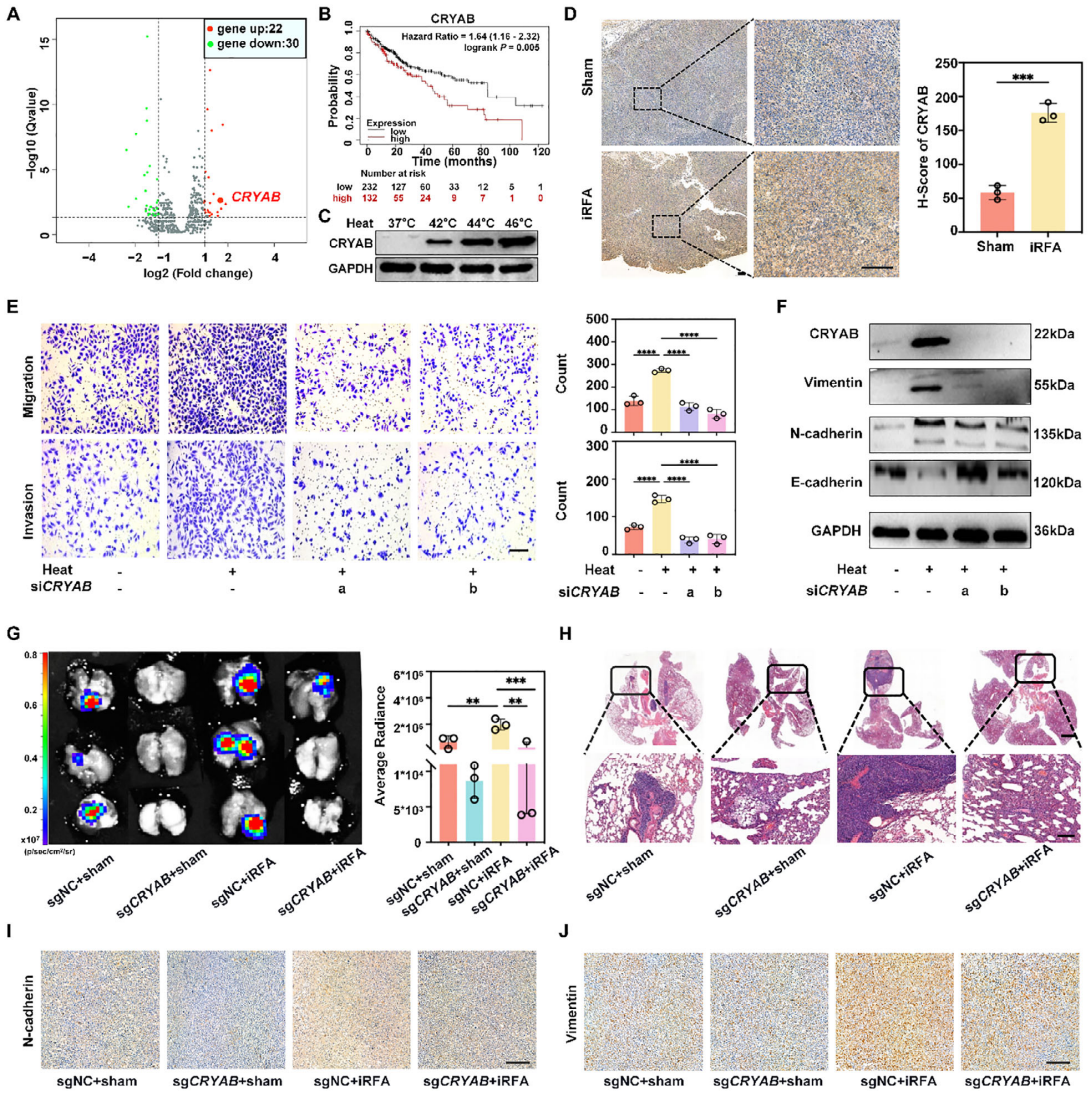
CRYAB is elevated in residual HCC post‐iRFA and drives EMT and metastasis. (A) Volcano plot of RNA‐seq analysis in HCC cells subjected to sublethal heat stress (44°C) versus control (37°C). CRYAB is the most significantly upregulated gene. (B) Kaplan–Meier overall survival analysis of HCC patients stratified by CRYAB expression (high vs. low) from the TCGA cohort. The number at risk is shown below. (C) Western blot of CRYAB protein in HCC cells treated at 37°C, 42°C, 44°C, and 46°C (*n* = 3). (D) IHC staining and H‐score quantification of CRYAB in residual tumor tissues following iRFA or sham operation (*n* = 3). Scale bar: 200 µm. (E) Transwell migration and invasion assays and quantification of heat‐stressed HCC cells with or without CRYAB knockdown (si*CRYAB*‐a, si*CRYAB*‐b) (*n* = 3). Scale bar: 100 µm. (F) Western blot analysis of CRYAB, Vimentin, E‐cadherin, and N‐cadherin in heat‐stressed HCC cells with or without CRYAB knockdown (si*CRYAB*‐a, si*CRYAB*‐b) (*n* = 3). (G) Ex vivo bioluminescence imaging of lung metastasis and average radiance quantification (n = 3). (H) Representative H&E staining of lung tissues of metastatic nodules from mice injected with heat‐stressed HCC cells carrying sgNC or sg*CRYAB* (*n* = 3). (I, J) IHC staining of N‐cadherin and Vimentin in subcutaneous residual tumor tissues from sgNC+sham, sgNC+iRFA, sg*CRYAB*+sham, and sg*CRYAB*+iRFA groups (*n* = 3). Scale bar: 200 µm. (Data are means ± SD. Calculated by Student's *t*‐test or One‐way ANOVA. ^*^ *p* < 0.05, ^**^ *p* < 0.01, ^***^ *p* < 0.001, ^****^ *p* < 0.0001).

To probe the cooperative regulatory network between residual cancer cells and TANs, we performed RNA sequencing (RNA‐seq) on HCC cells subjected to sublethal heat stress (44°C) as established in our prior work to mimic iRFA treatment in vitro [10]. Among the 52 differentially expressed genes (DEGs) identified by volcano plot and heatmap analyses, CRYAB was the most significantly upregulated transcript (Figure 1A; Figure S1A). The ranking of the top 6 upregulated genes by log2FC and significance is shown in Figure S1B. Clinical correlation analysis showed that CRYAB expression was significantly higher in HCC tissues than in adjacent normal tissues, and Kaplan–Meier survival analysis indicated that high CRYAB expression was closely associated with poor overall survival (Figure 1B), suggesting a critical role of CRYAB in HCC progression and prognosis. CRYAB expression was markedly elevated in heat‐stressed HCC cells and consistently high in residual HCC tissues after iRFA (Figure 1C,D; Figure S1C,D). In vitro, transwell assays demonstrated that heat stress increased the migration and invasion of HCC cells by 2.03‐fold and 1.96‐fold, respectively; these pro‐metastatic effects were significantly reversed by CRYAB knockdown (Figure 1E; Figure S1E). Western blot analysis further confirmed that heat stress induced EMT in HCC cells, as evidenced by upregulated Vimentin and N‐cadherin and downregulated E‐cadherin expression, and CRYAB knockdown effectively reversed this phenotypic switch (Figure 1F; Figure S1F).

To investigate whether CRYAB expression regulates residual cancer metastasis post‐iRFA, we injected heat‐stressed HCC cells with CRYAB knockdown (sg*CRYAB*) or negative control (sgNC) via the tail vein of mice. Ex vivo IVIS imaging showed that iRFA promoted lung metastasis of HCC cells, whereas sg*CRYAB* group markedly suppressed this effect (Figure 1G). H&E staining of lung tissues further confirmed fewer metastatic nodules in the sg*CRYAB* + iRFA group compared with the sgNC + iRFA group (Figure 1H). Immunohistochemistry (IHC) staining of residual cancer tissues also revealed that iRFA activated N‐cadherin and Vimentin expression expression; these changes were significantly reversed upon CRYAB knockdown (Figure 1I,J). These data consistently demonstrate that CRYAB acts as a thermal stress sensor that translates sublethal heat input into pro‐metastatic signaling and governs the metastatic potential of residual HCC cells post‐iRFA by promoting EMT.

### 3.2 CRYAB Promotes N2 Polarization of TANs in the Residual HCC Microenvironment post‐iRFA

**FIGURE 2:**
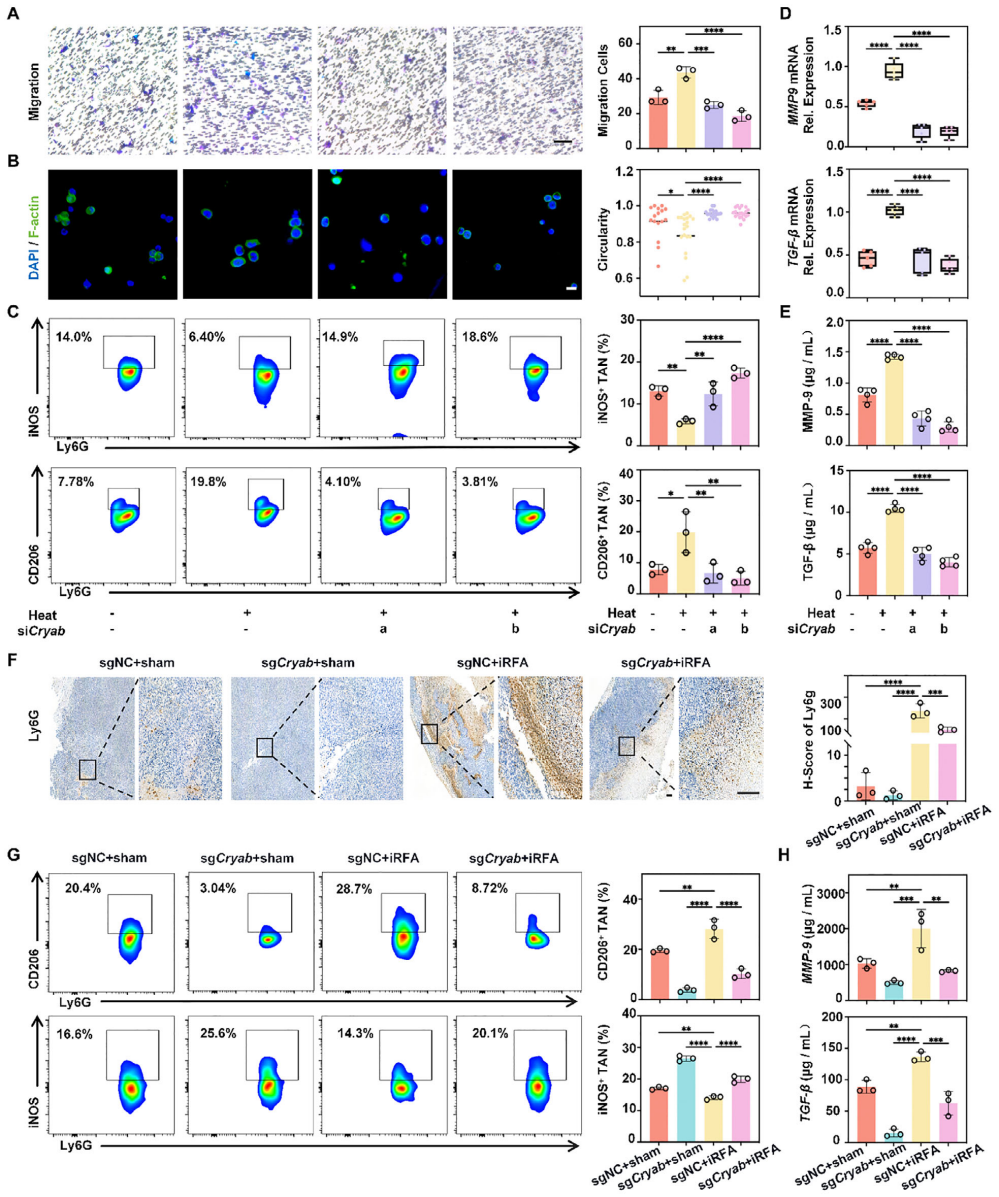
CRYAB promotes N2 polarization of TANs in the residual HCC microenvironment post‐iRFA. (A) Chemotaxis assay and quantification showing migration of bone marrow‐derived neutrophils toward conditioned medium (TCM) from heat‐stressed HCC cells with sgNC or sg*Cryab* (*n* = 3). Scale bar: 50 µm. (B) Representative immunofluorescence images (DAPI/F‐actin) of TANs cultured with the indicated TCM (*n*  =  3). Scale bar: 10 µm. Circularity quantification reflects morphological changes (spindle‐like N2 vs. rounded N1). (C) Flow cytometry (FCM) analysis and quantification of CD206^+^ TANs and iNOS^+^ TANs in the indicated TCM treatment groups (*n*  =  3). (D) qPCR analysis of cytokine expression (MMP‐9 and TGF‐β) in heat‐stressed HCC cells with sgNC or sg*Cryab* (*n*  =  5). (E) ELISA quantification of TGF‐β and MMP‐9 secretion in the indicated TCM treatment groups (*n*  =  4). (F) IHC staining for Ly6G and CRYAB and H‐score quantification in subcutaneous residual tumors from sgNC+sham, sgNC+iRFA, sg*Cryab*+sham, and sg*Cryab*+iRFA groups (*n*  =  3). Scale bar: 200 µm. (G) FCM analysis and percentages of Ly6G^+^ TANs co‐stained with CD206 and iNOS from the four groups (*n*  =  3). (H) ELISA quantification of TGF‐β and MMP‐9 in the tumor microenvironment of the four groups (*n*  =  3). (Data are means ± SD. Calculated by One‐way ANOVA. ^*^ *p* < 0.05, ^**^ *p* < 0.01, ^***^ *p* < 0.001, ^****^ *p* < 0.0001).

Our prior work demonstrated that residual tumor after iRFA recruits abundant TANs and drives them toward an N2 polarization, exacerbating the immunosuppressive TME [11]. However, whether CRYAB participates in this process remains unresolved. To systematically investigate the effect of CRYAB on TANs phenotype and function, we established a co‐culture system using bone marrow‐derived neutrophils and tumor conditioned medium (TCM) from heat‐stressed HCC cells with or without CRYAB knockdown in vitro. Chemotaxis assays showed that TCM from heat‐stressed HCC cells enhanced TAN migration 1.48‐fold relative to control, whereas CRYAB knockdown had the opposite effect (Figure 2A). Morphologically, TANs exposed to the TCM of heat‐stressed HCC cells adopted a spindle‐like N2 appearance, whereas CRYAB knockdown restored a rounded N1 morphology (Figure 2B). Flow cytometry (FCM) further showed that the percentage of CD206^+^ TANs in the heat stress group (19.87% ± 6.60%) was significantly higher than that in the CRYAB knockdown group (5.03% ± 2.20%); conversely, the proportion of iNOS^+^ TANs was significantly elevated in the CRYAB knockdown group (Figure 2C). Therefore, we speculated that CRYAB knockdown downregulates certain cytokines to reverse TANs chemotaxis and polarization. Using ‌quantitative PCR (qPCR), we screened essential cytokines responsible for these effects on TANs, including granulocyte colony‐stimulating factor (G‐CSF), interleukin‐6 (IL‐6), Chemokine (C‐X‐C motif) ligand 1 (CXCL1), TGF‐β, and MMP‐9, and found that TGF‐β and MMP‐9 mRNA levels were the most significantly decreased in heat‐stressed HCC cells upon CRYAB knockdown (Figure 2D; Figure S2A,B). ELISA further verified that the secretion of TGF‐β and MMP‐9 was reduced in the CRYAB knockdown group (Figure 2E). These results suggest that heat‐stressed HCC cells may drive N2 polarization of TAN through CRYAB‐mediated upregulation of TGF‐β and MMP‐9.

To further validate the role of CRYAB in regulating TANs in vivo, we employed a subcutaneous iRFA residual cancer model, and the mice were divided into four groups: sgNC control, sgNC + iRFA, sg*Cryab*, and sg*Cryab* + iRFA. Tumor tissues were harvested 21 days after iRFA. Gross images and tumor volume curves indicated that CRYAB knockdown significantly inhibited iRFA‐induced residual HCC recurrence (Figure S3A–C). IHC staining showed obviously increased TANs infiltration in residual cancer tissues of the iRFA group, whereas TANs accumulation was reduced in the sg*Cryab* + iRFA group (Figure 2F). FCM further confirmed that iRFA upregulated CD206 and downregulated iNOS in TANs, suggesting CRYAB knockdown significantly promoted N1 TAN polarization in residual HCC (Figure 2G). ELISA results also showed that TGF‐β and MMP‐9 levels were markedly increased in the TME after iRFA, and these effects were effectively inhibited in the sg*Cryab* group (Figure 2H). Further FCM results also indicated that CRYAB knockdown significantly increased infiltration of CD4^+^ and CD8^+^ T cells and decreased Treg accumulation post‐iRFA (Figure S3D). Collectively, the above results indicate that iRFA not only leaves residual tumors but also promotes malignant progression by remodeling both tumor cell phenotypes and the immunosuppressive TME, with CRYAB serving as a crucial regulator of TAN N2 polarization. Inhibiting CRYAB not only reverses residual cancer EMT but also suppresses N2 TAN polarization and activates anti‐tumor immunity.

### 3.3 CRYAB Stabilizes β‐Catenin by Suppressing Ubiquitin‐Dependent Degradation to Promote EMT and N2 Polarization

**FIGURE 3:**
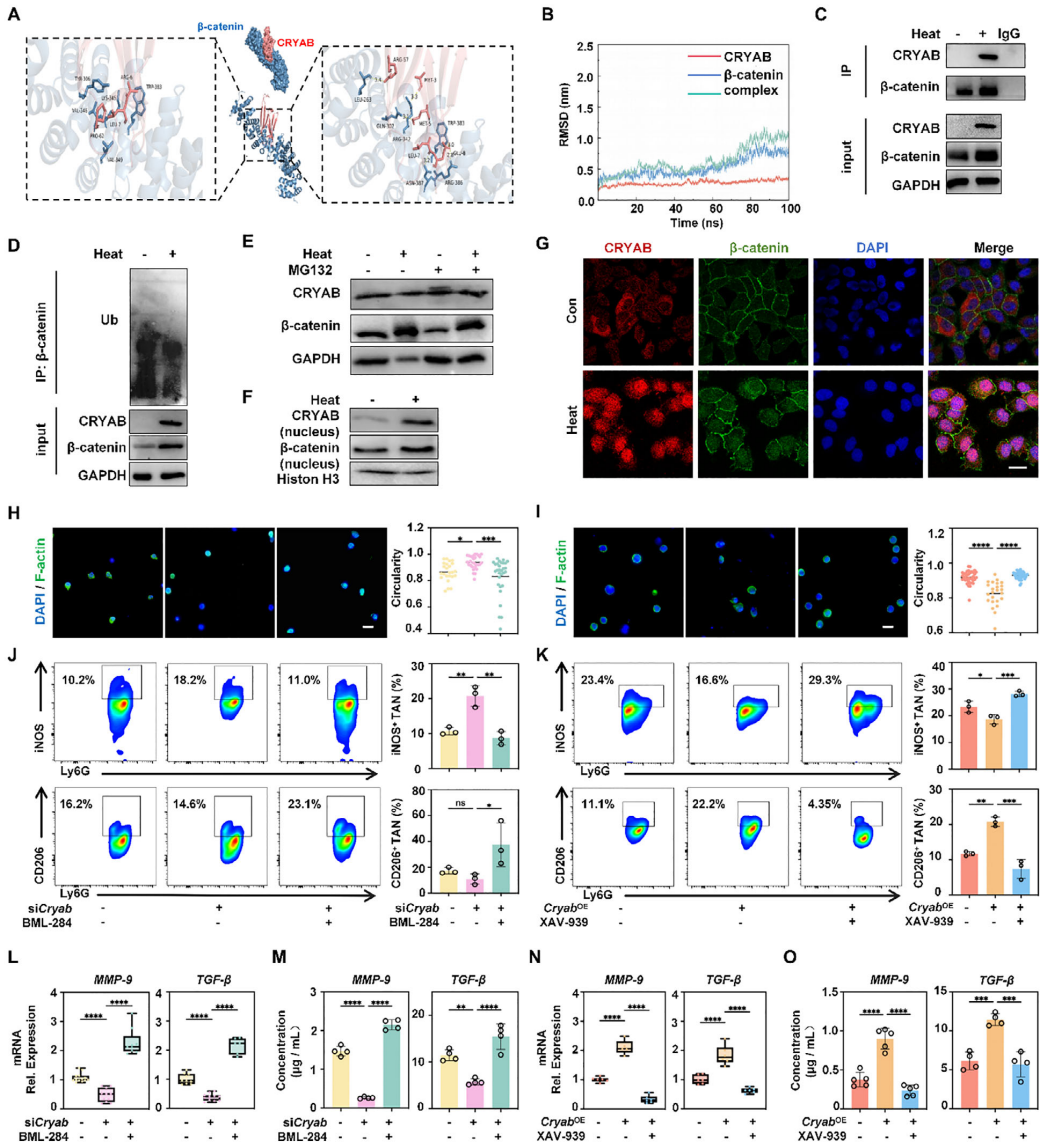
CRYAB stabilizes β‐catenin by suppressing ubiquitin‐dependent degradation to promote EMT and N2 polarization. (A) Molecular docking model showing the binding interface between CRYAB (red) and β‐catenin (blue). Hydrophobic interactions (left, residues LEU7, PRO62, VAL349, ARG6, ARG57) and hydrogen bonds (right, residues ARG342, GLN302, ASN387, ARG386, TRP383, LEU263) collectively stabilize the complex. (B) RMSD trajectories from 100‐ns molecular dynamics simulations, showing stable conformational dynamics for both CRYAB (∼0.35 nm) and β‐catenin (∼0.80 nm), confirming a robust and stable interaction interface. (C) Co‐IP of β‐catenin with CRYAB in control and heat‐stressed cells (*n*  =  3). (D) Ubiquitination assay (IP: β‐catenin; IB: Ub) showing reduced β‐catenin ubiquitination upon heat stress (*n*  =  3). (E) Western blot of β‐catenin in control and heat‐stressed HCC cells treated with MG132 (proteasome inhibitor) or vehicle (*n*  =  3). (F) Nuclear/cytoplasmic fractionation and western blot for CRYAB and β‐catenin in heat‐stressed HCC cells. Histone H3 serves as a nuclear marker (*n*  =  3). (G) Immunofluorescence image of nuclear CRYAB and β‐catenin in control and heat‐stressed HCC cells (n  =  3). Scale bar: 50 µm. (H) Representative immunofluorescence images and quantification of TAN morphology cultured with si*Cryab* and BML‐284 treatment (*n*  =  3). (I) Representative immunofluorescence images and quantification of TAN morphology cultured with *Cryab^OE^* and XAV‐939 treatment (*n*  =  3). Scale bar: 10 µm. (J) FCM analysis and quantification of iNOS^+^ TANs and CD206^+^ TANs cultured with si*Cryab* and BML‐284 treatment (*n*  =  3). (K) FCM analysis and quantification of iNOS^+^ TANs and CD206^+^ TANs cultured with *Cryab^OE^* and XAV‐939 treatment (*n*  =  3). (L–O) qPCR (*n*  =  5) and ELISA (*n*  =  4) analyses of TGF‐β and MMP‐9 expression and secretion in the si*Cryab*+BML‐284 and *Cryab^OE^* +XAV‐939 rescue systems, respectively. (Data are means ± SD. Calculated by Student's *t*‐test or One‐way ANOVA. ^*^ *p* < 0.05, ^**^ *p* < 0.01, ^***^ *p* < 0.001, ^****^ *p* < 0.0001).

To dissect the mechanism by which CRYAB governs EMT in residual HCC cells and N2 polarization in TANs, we focused on the analysis of critical signaling mediators. RNA‐seq and STRING analysis collectively identified β‐catenin (CTNNB1) as a potential CRYAB‐interacting partner (Figure S4A,B). To define the structural basis of the CRYAB–β‐catenin interaction, we performed molecular docking and MD simulations. Molecular docking identified a high‐affinity binding model with a docking score of −267.80 and a confidence score of 0.9134 (Table S1). Interaction analysis demonstrated that β‐catenin is primarily recognized through its ARM repeat domain, with both hydrogen bonding and hydrophobic interactions contributing to the binding interface (Figure 3A). Energy decomposition further identified key contributing residues within the ARM domain and the CRYAB interaction surface (Figure S4C,D). MD simulations showed stable RMSD for both proteins with consistent hydrogen bonding, confirming a robust interaction interface (Figure 3B). Besides, MD simulation revealed a binding free energy of −705.30 kJ/mol, confirming a stable direct interaction without the requirement of bridging proteins (Figure S4E). These computational data demonstrate that CRYAB directly binds to β‐catenin through the ARM repeat domain, providing a structural basis for its chaperone‐mediated stabilization.

Further western blot assay revealed that heat stress increased β‐catenin protein expression in HCC cells. However, qPCR analyses showed that β‐catenin mRNA levels remained unchanged (Figure S5A–C), suggesting that CRYAB regulates β‐catenin at the post‐translational rather than transcriptional level. As a molecular chaperone, CRYAB primarily functions to bind target proteins and protect them from proteolytic degradation [19]. To determine whether CRYAB interacts with β‐catenin, we performed co‐immunoprecipitation (Co‐IP) and confirmed that CRYAB directly bound to β‐catenin after heat stress (Figure 3C). Ubiquitination assays further revealed that heat‐stress‐induced upregulation of CRYAB inhibited ubiquitination of β‐catenin, thereby enhancing its protein stability (Figure 3D). To verify whether this stabilization occurs through the ubiquitin–proteasome pathway, we treated cells with the proteasome inhibitor MG132 [31]. The results showed MG132 significantly upregulated β‐catenin in heat‐stressed cells but not in control cells (Figure 3E), suggesting that β‐catenin stability is mainly regulated by the ubiquitin–proteasome pathway after heat stress. We next examined the subcellular localization of CRYAB and β‐catenin by immunofluorescence staining, which revealed nuclear translocation of both proteins in heat‐stressed HCC cells. Nuclear/cytoplasmic fractionation followed by Western blot further verified their increased nuclear accumulation upon heat stress (Figure 3F,G). These data demonstrate that CRYAB directly binds β‐catenin and protects it from ubiquitin‐proteasome‐mediated degradation, thereby promoting β‐catenin stabilization and nuclear translocation in response to heat stress.

To determine whether CRYAB‐mediated regulation of EMT and TAN polarization after heat stress depends on β‐catenin activation, we employed BML‐284 [32], a β‐catenin agonist that activates Wnt/β‐catenin signaling, and XAV‐939 [33], a β‐catenin inhibitor that promotes β‐catenin degradation by stabilizing the AXIN destruction complex. To determine the appropriate concentration for mechanistic studies, we evaluated the cytotoxicity of XAV‐939 against HCC cells using the CCK‐8 assay. The IC_50_ of XAV‐939 was determined to be 45.5 µm (Figure S5D). Based on this result and consistent with previous reports, a concentration of 10 µM was selected for subsequent in vitro mechanistic experiments. This concentration is well below the IC_50_ and maintains cell viability, thereby excluding direct cytotoxicity and allowing specific evaluation of Wnt/β‐catenin pathway inhibition. Compared with the si*Cryab* group, the si*Cryab* + BML‐284 group promoted TAN polarization toward a spindle‐like morphology and increased the proportion of N2 TANs (Figure 3H,J ). In contrast, compared with the *Cryab^OE^* group, the *Cryab^OE^* + XAV‐939 group significantly suppressed EMT and promoted TAN polarization toward the N1 phenotype (Figure 3I,K ; Figure S5E). qPCR and ELISA confirmed that si*Cryab* + BML‐284 restored heat‐induced mRNA expression and secretion of MMP‐9 and TGF‐β, whereas Cryab^OE^ + XAV‐939 significantly reversed these effects (Figure 3L–O). Treating neutrophils with a TGF‐β inhibitor (SB‐431542) or MMP‐9 inhibitor (SB‐3CT) markedly abolished the N2‐polarizing effect of conditioned medium from heat‐stressed or *Cryab^OE^* HCC cells, accompanied by elevated iNOS and reduced CD206 (Figure S5F,G). These data confirm that TGF‐β and MMP‐9 serve as critical downstream mediators for CRYAB‐induced N2 TAN polarization in the post‐iRFA microenvironment. IHC staining of residual cancer tissues also revealed that iRFA activated β‐catenin expression, while the change was significantly reversed upon CRYAB knockdown (Figure S5H). Together, these results define CRYAB as a thermal‐biological transducer that stabilizes β‐catenin to drive nuclear translocation, thereby linking thermal stress to the immune‐metastatic cascade by promoting EMT in residual HCC cells and N2 polarization of TANs post‐iRFA.

### 3.4 The CRYAB/Βcatenin‐ Axis‐ Promotes‐ CTC Cluster‐ Formation‐ Between Residual Cancer Cells‐ and TANs

**FIGURE 4:**
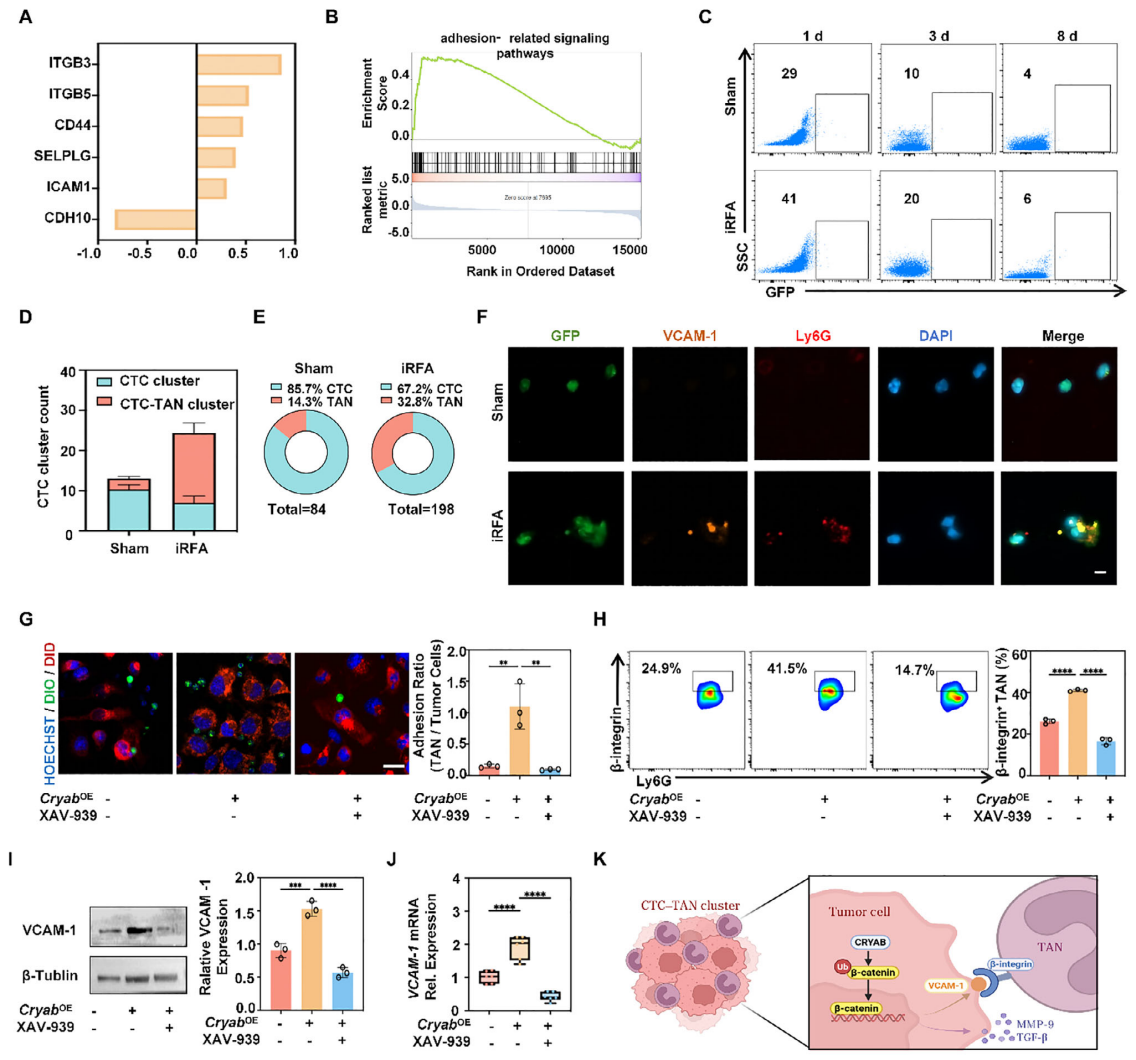
The CRYAB/β‐catenin axis promotes CTC cluster formation between residual cancer cells and TANs. (A) Heatmap of differentially expressed cell adhesion‐related genes from RNA‐seq of heat‐stressed HCC cells. (B) Gene set enrichment analysis (GSEA) plot showing enrichment of adhesion‐related signaling pathways in TANs sorted from post‐iRFA residual tumors. (C) FACS plots showing GFP^+^ CTCs (HCC cells) in peripheral blood at 1, 3, and 8 days after iRFA or sham operation (*n* = 3). (D) Number of CTC clusters (Ly6G‐ GFP+) and CTC–TAN (Ly6G+ GFP+) clusters per 1 mL of peripheral blood (*n* = 3). (E) Pie charts showing the mean percentage of CTC clusters (green) and CTC–TAN clusters (red) in 3 mice per group. (F) Representative images of VCAM‐1^+^ CTC‐TAN cluster. GFP (cancer cells), green; VCAM‐1, orange; Ly6G (TAN), red (*n* = 3). Scale bar:10 µm. (G) Representative fluorescence images and quantification of CTC cluster formation in co‐cultures of heat‐stressed HCC cells and TANs with *Cryab^OE^* and XAV‐939 treatment (*n* = 3). Scale bar: 30 µm. (H) FCM analysis and quantification of β‐integrin^+^ TANs with *Cryab^OE^* and XAV‐939 treatment (*n* = 3). (I) Western blot and quantification of VCAM‐1 in HCC cells with *Cryab^OE^* and XAV‐939 treatment (*n* = 3). (J) qPCR quantification of VCAM‐1 mRNA in HCC cells with *Cryab^OE^* and XAV‐939 treatment (*n* = 6). (K) Schematic illustration of the CRYAB/β‐catenin axis driving CTC cluster formation through enhanced VCAM‐1/β‐integrin binding. (Data are means ± SD. Calculated by Student's *t*‐test or One‐way ANOVA. ^*^ *p* < 0.05, ^**^ *p* < 0.01, ^***^ *p* < 0.001, ^****^ *p* < 0.0001).

Given that mesenchymal‐like tumor cells and N2‐polarized TANs are prone to form heterogeneous CTC–immune cell clusters [16], we investigated whether the CRYAB/β‐catenin axis facilitates the formation of such clusters between residual cancer cells and TANs. First, analysis of RNA‐seq data of residual HCC cells revealed significant upregulation of adhesion‐related genes (Figure 4A). In addition, proteomic profiling of flow‐sorted TANs from post‐iRFA residual tumors and GSEA revealed robust activation of adhesion signaling pathways (Figure 4B). To assess the impact of iRFA on CTC cluster formation in vivo, we collected peripheral blood samples from mice before and after ablation and performed CTC cluster enumeration. The count of peripheral blood CTCs peaked at 1 day after iRFA, increasing by 1.52‐fold compared with pre‐ iRFAoperation, and slightly decreased at 3 and 8 days (Figure 4C; Figure S6A). Besides, further quantitative analysis revealed that control mice harbored 12.67 ± 1.53 CTC clusters per mL of blood, of which 2.67 ± 0.58 were heterotypic CTC‐ TAN clusters. In contrast, the iRFA group exhibited a significantly higher number of CTC clusters (24.33 ± 1.53 per mL), with 17.33 ± 2.52 being CTC–TAN clusters (Figure 4D). The relative proportion of TANs within CTC clusters increased substantially from 14.3% in controls to 32.8% after iRFA (Figure 4E), indicating that ablation promotes heterotypic cluster formation. To directly visualize these clusters, GFP^+^ CTCs were immunostained with anti‐VCAM‐1 and anti‐Ly6G antibodies. Confocal imaging further confirmed that VCAM‐1 expression was markedly upregulated on CTCs in the iRFA group, with tight adherence between VCAM‐1^+^ GFP^+^ CTCs and Ly6G^+^ TANs in iRFA samples, whereas such clusters were rarely observed in controls (Figure 4F).

In co‐culture assays, *Cryab^OE^* group further increased the CTC cluster binding ratio, whereas XAV‐939 treatment reduced cluster formation (Figure 4G). Studies have shown that vascular cell adhesion molecule‐1 (VCAM‐1) and β‐integrin constitute a receptor–ligand pair that may be implicated in the formation of CTC clusters in tumors [34, 35]. In our results, FCM and qPCR confirmed that *Cryab^OE^*‐TCM increased β‐integrin on TANs, and the effect was markedly reversed by XAV‐939 treatment (Figure 4H). Western blot and qPCR further showed that the *Cryab^OE^* + XAV‐939 group significantly suppressed VCAM‐1 expression on HCC cells by 1.7‐fold compared with the *Cryab^OE^* group (Figure 4I‐J). Collectively, these results indicate that heat stress promotes CTC cluster formation via the CRYAB/β‐catenin axis by enhancing VCAM‐1/β‐integrin interactions between residual HCC cells and TANs (Figure 4K), thereby providing “high‐efficiency metastatic seeds” for HCC metastasis after iRFA.

### 3.5 Synthesis and Characterization of MMP‐9‐Responsive Thermosensitive Drug‐Loaded Hydrogel‐ (XB@Gel), Optimizing‐ Tumor‐ Targeting‐ Post–iRFA

**FIGURE 5:**
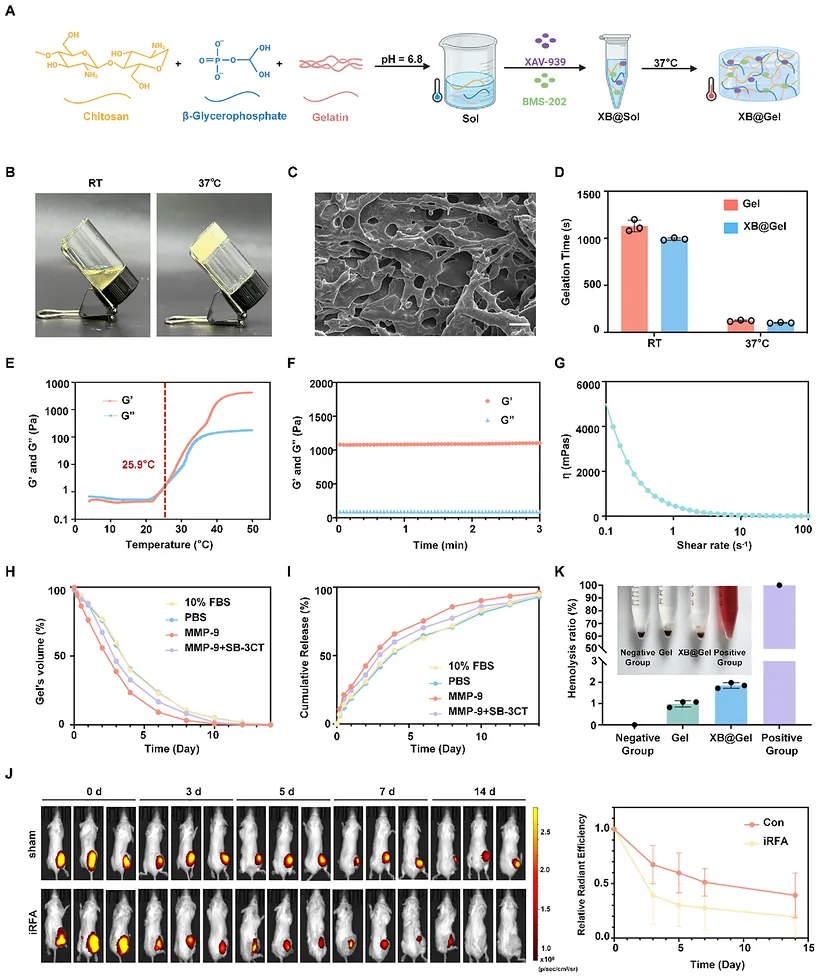
Synthesis and characterization of MMP‐9‐responsive thermosensitive drug‐loaded hydrogel (XB@Gel). (A) Schematic illustration of XB@Gel preparation and its sol–gel transition at 37°C. (B) Photographs of XB@Gel as a flowable solution at room temperature (RT) and as a gel at 37°C. (C) SEM image of lyophilized XB@Gel showing its porous interconnected microstructure. Scale bar: 20 µm. (D) Gelation time of blank gel and XB@Gel at RT and 37°C (*n* = 3). (E) Rheological temperature sweep showing gelation temperature (G′–G″ crossover) at 25.9°C. (F) Time sweep at 37°C demonstrating stable gel formation (G′ > G″). (G) Viscosity (η) as a function of shear rate, illustrating shear‐thinning behavior. (H) In vitro degradation profiles of the hydrogels under the indicated treatment conditions. (I) Cumulative release profiles of FITC under the indicated treatment conditions over 14 days. (J) Representative IVIS images and quantitative analysis of ICG@Gel retention in sham and iRFA mice (*n* = 3). Data were normalized to the value on day 0 for each mouse and are presented as mean ± SD. (K) Hemolysis assay of XB@Gel and blank gel compared with negative and positive controls. Inset: photograph of centrifuged erythrocyte suspensions (*n* = 3). Data are means ± SD.

While XAV‐939 blocks the CRYAB/β‐catenin axis and inhibits CTC generation, systemic application shares the key clinical drawbacks of chemotherapy, including insufficient local retention in residual tumors and potential off‐target effects [36]. Given the pathologically elevated levels of MMP‐9 in the post‐iRFA microenvironment, we developed an MMP‐9‐responsive thermosensitive hydrogel (XB@Gel) for co‐delivery of XAV‐939 and BMS‐202 via a simple physical mixing method. By incorporating gelatin into the chitosan/β‐glycerophosphate (β‐GP) system, the hydrogel was rationally optimized to achieve MMP‐9 responsiveness and favorable mechanical performance (Figure 5A). Our results indicated that increasing gelatin content enhanced hydrogel mechanical strength, while 0.5% gelatin provided the optimal balance of drug release, shear‐thinning properties, and injectability (Figure S7A–D). As shown in Figure 5B, XB@Gel was a pale‐yellow solution at room temperature and underwent a reversible sol–gel transition upon warming to physiological temperature (37°C). SEM imaging of lyophilized XB@Gel revealed a typical porous and interconnected microstructure (Figure 5C). The gelation time at 37°C was shortened to 102 ± 5 s, compared with 992 ± 13 s at room temperature, and the addition of XAV‐939 and BMS‐202 had no significant effect on gelation kinetics (Figure 5D). Dynamic rheometry showed that the gelation temperature of XB@Gel was 25.9°C (Figure 5E). At 37°C, the storage modulus (G') was significantly higher than the loss modulus (G'') and remained stable, indicating reliable in situ gelation capacity (Figure 5F). The viscosity of XB@Gel decreased with increasing shear rate, demonstrating shear‐thinning behavior and favorable injectability for clinical application (Figure 5G). The encapsulation efficiency of XB@Gel was 89.93 ± 3.51% (Figure S7E). XB@Gel could be continuously injected through a 24G needle into PBS solution at 37°C, demonstrating excellent injectability (Figure S7F). Degradation and drug release assays showed that MMP‐9 accelerated the degradation of XB@Gel compared with the PBS group; this acceleration was largely abolished by SB‐3CT, while 10% FBS proteases did not trigger obvious degradation (Figure 5H). In vitro drug release profiles showed that MMP‐9 induced sustained drug release for up to 14 days (Figure 5I). In vivo experiments also confirmed that XB@Gel could be retained in mouse subcutaneous tissues for at least 14 days (Figure S7G). IVIS imaging indicated that the iRFA group exhibited significantly faster fluorescence decay than the control group, suggesting that elevated MMP‐9 in the iRFA microenvironment accelerates local drug release in vivo (Figure 5J). Biocompatibility evaluation showed that the hemolysis rate of XB@Gel was 1.8% (Figure 5K). H&E staining and serological tests confirmed no obvious systemic toxicity of XB@Gel (Figure S8). In summary, by exploiting the pathologically elevated MMP‐9 in the post‐iRFA microenvironment as a local trigger, we successfully developed a dual‐responsive, biodegradable hydrogel that integrates thermosensitive in situ gelation, sustained on‐demand drug release, local MMP‐9 depletion, and excellent biosafety. This system constitutes an efficient platform for localized drug delivery tailored to the unique challenges of residual HCC after RFA.

### 3.6 XB@Gel Suppresses Post‐iRFA Tumor Recurrence by Blocking the CRYAB/β‐Catenin Axis

**FIGURE 6:**
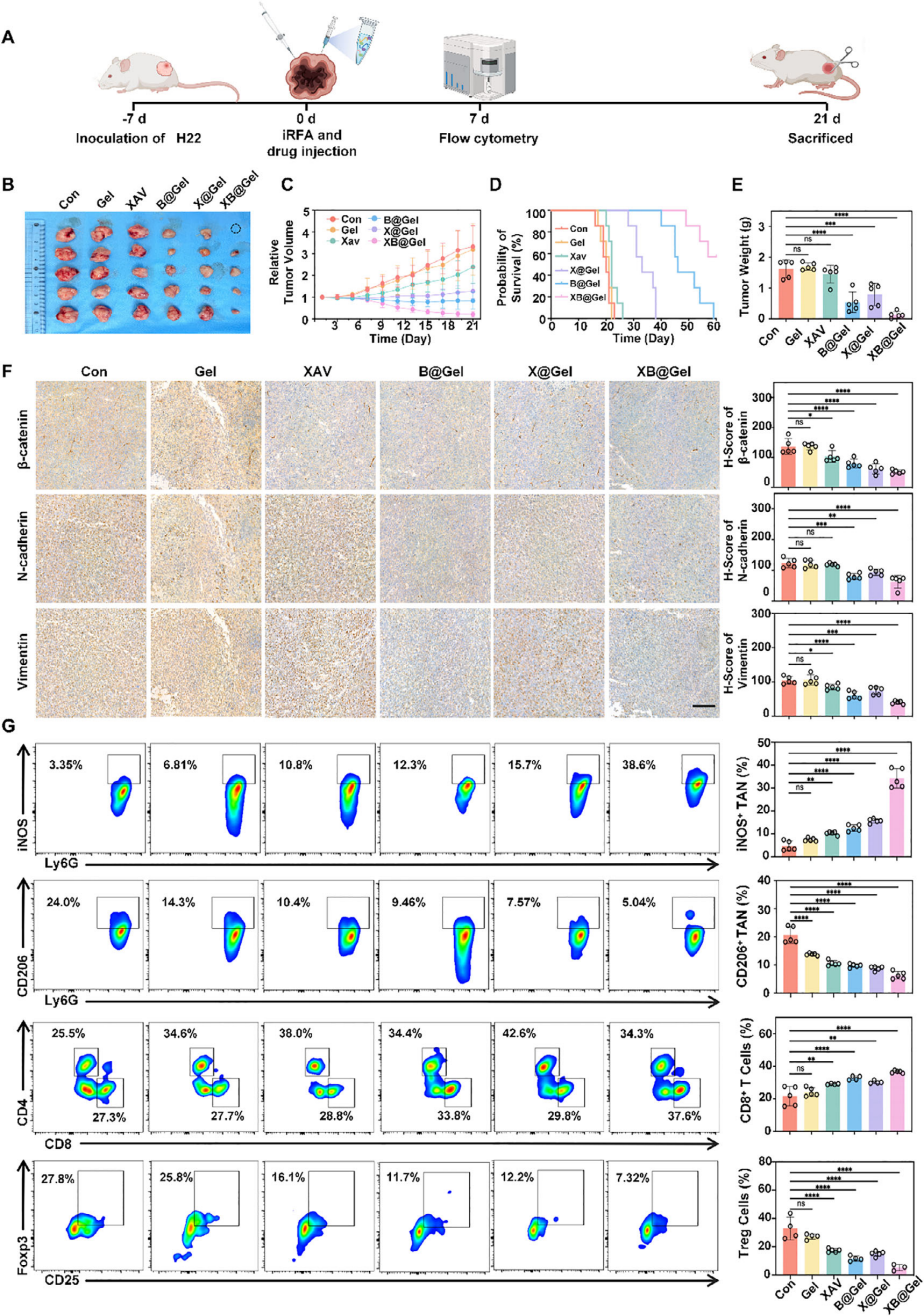
XB@Gel suppresses post‐iRFA tumor recurrence by blocking the CRYAB/β‐catenin axis. (A) Flowchart of the animal model illustrating the treatment timeline in a subcutaneous tumor. Mice were treated with PBS, Gel, free XAV‐939 (XAV), BMS‐202@Gel (B@Gel), XAV‐939@Gel (X@Gel), and XB@Gel following RFA (*n*  =  5). (B) Comparative analysis of tumor sizes under various post‐RFA treatment conditions (*n*  =  5). (C) Growth curve of the HCC residual tumor by caliper after different treatments (*n*  =  5). (D) Line graph of survival rates in tumor‐bearing mice under different treatments (*n*  =  7). (E) Histogram comparing tumor weights across treatment groups (*n*  =  5). (F) IHC staining for β‐catenin, N‐cadherin, and Vimentin in residual tumor tissues after different treatments (*n*  =  5). Scale bar: 200 µm. (G) FCM analysis and quantification of CD206^+^ TANs, iNOS^+^ TANs, Tregs, and CD8^+^ T cells within the residual tumor microenvironment under different treatments (*n*  =  5). (Data are means ± SD. Calculated by Student's *t*‐test or One‐way ANOVA. ^*^ *p* < 0.05, ^**^ *p* < 0.01, ^***^ *p* < 0.001, ^****^ *p* < 0.0001).

To evaluate the anti‐recurrence efficacy of XB@Gel, we used the subcutaneous iRFA residual cancer model and randomly divided mice into six groups: PBS (control), blank hydrogel (Gel), free XAV‐939 (XAV), BMS‐202@Gel (B@Gel), XAV‐939@Gel (X@Gel), and XAV‐939/BMS‐202@Gel (XB@Gel). Treatments were administered locally immediately after iRFA, and tumor volume was monitored serially (Figure 6A). Tumor growth curves and tumor weight data showed that tumor burden increased markedly in the control and Gel groups by day 21 post‐operation. The XAV, B@Gel, and X@Gel groups exhibited moderate inhibitory effects, while tumor recurrence was significantly suppressed in the XB@Gel group (Figure 6B,C,E). During the 60‐day observation period, median survival was not reached in the XB@Gel group, and the overall survival rate at the end of the study was 71.4% (Figure 6D). This survival outcome was significantly better than that of all other groups. IHC staining confirmed that the expression levels of β‐catenin, N‐cadherin, and Vimentin were significantly reduced in residual cancer tissues of the XB@Gel group, indicating marked reversal of EMT (Figure 6F). FCM analysis showed that, relative to control, the infiltration of CD206^+^ TANs and Tregs in the residual cancer microenvironment of the XB@Gel group decreased from 21.18 ± 3.18% and 32.93 ± 8.06% to 6.63 ± 1.19% and 5.46 ± 2.06%, respectively. Meanwhile, iNOS^+^ TANs and CD8^+^ T cells increased from 4.91 ± 1.89% and 21.70 ± 5.93% to 33.10 ± 4.72% and 36.62 ± 0.77% (Figure 6G). These data demonstrate that local release of XAV‐939 and BMS‐202 from XB@Gel synergistically blocks CRYAB/β‐catenin axis‐driven EMT and N2 TAN polarization, simultaneously reversing the mesenchymal traits of residual cancer cells and the immunosuppressive TME, thereby efficiently inhibiting residual HCC recurrence after iRFA.

### 3.7 XB@Gel Inhibits Residual HCC Metastasis after iRFA by Blocking the CRYAB/Β‐Catenin Axis

**FIGURE 7:**
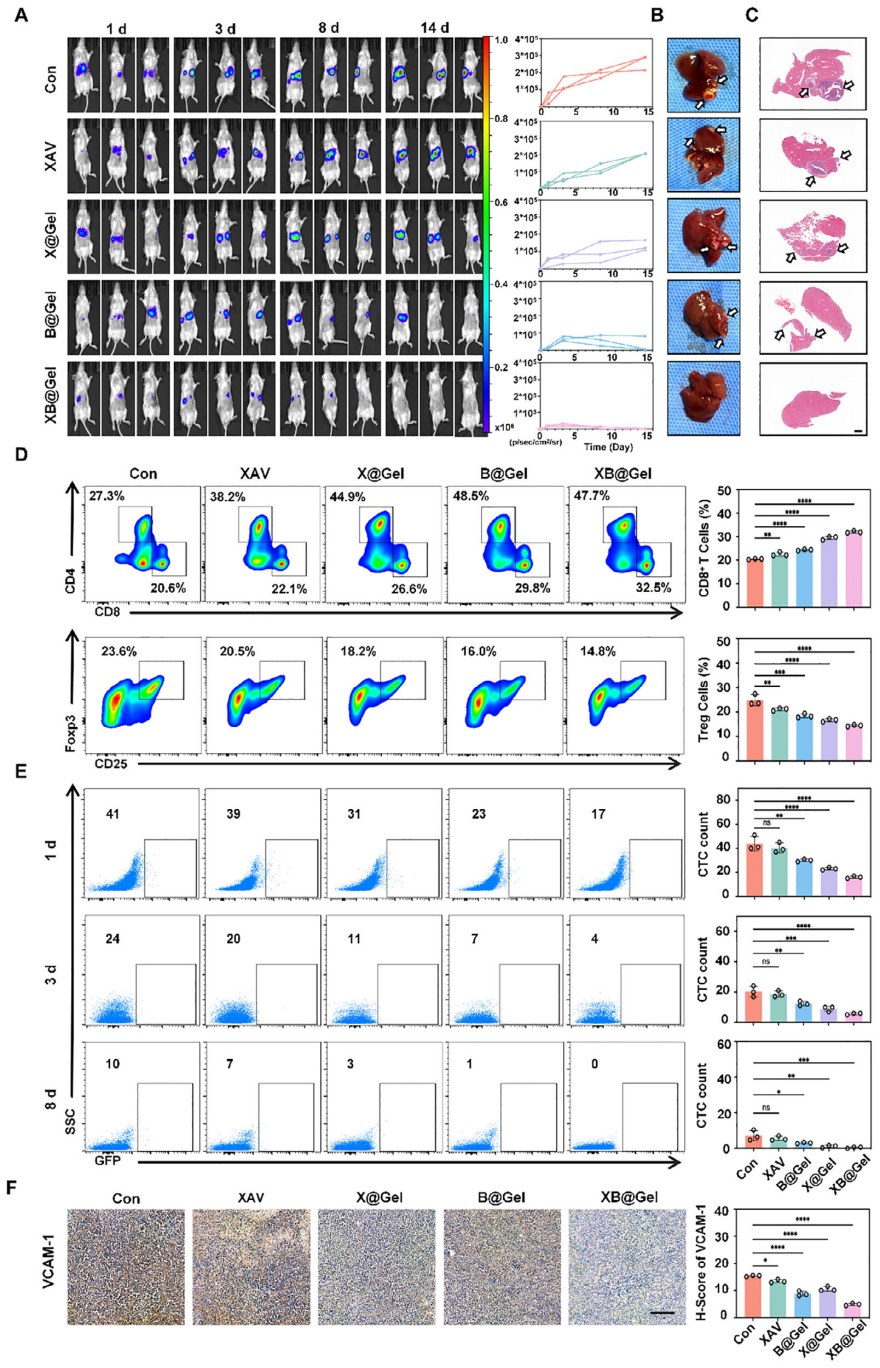
XB@Gel inhibits residual HCC metastasis after iRFA by blocking the CRYAB/β‐catenin axis. (A) In vivo bioluminescence imaging and quantification of intrahepatic metastasis at day 14 post‐iRFA in the five treatment groups: control, free XAV‐939, B@Gel, X@Gel, and XB@Gel (*n*  =  3). (B, C) Representative gross liver images and H&E‐stained liver sections showing metastatic nodules (*n*  =  3). Scale bar: 1000 µm. (D) FCM analysis and quantification of CD8^+^ T cells and Tregs in spleen tissues after different treatments (*n*  =  3). (E) FCM analysis showing CTC numbers (GFP^+^ HCC cells) in peripheral blood at 1, 3, and 8 days after different treatments (*n*  =  3). (F) IHC staining and H‐score quantification of VCAM‐1 in residual tumor tissues after different treatments (*n*  =  3). (Data are means ± SD. Calculated by Student's t‐test or One‐way ANOVA. ^*^ *p* < 0.05, ^**^ *p* < 0.01, ^***^ *p* < 0.001, ^****^ *p* < 0.0001).

To evaluate the anti‐metastatic efficacy of XB@Gel, we established a liver metastasis model by subcutaneous inoculation of H22 HCC cells, followed by intrasplenic injection of H22‐luc‐GFP cells 3 days later. On day 4, subcutaneous tumors were subjected to iRFA and local hydrogel treatment. Mice were assigned to five groups: control, free XAV‐939, B@Gel, X@Gel, and XB@Gel. In vivo bioluminescence imaging at day 14 post‐iRFA revealed extensive intrahepatic metastatic lesions and increased fluorescence intensity in the control and free XAV groups. Metastatic lesions were slightly reduced in the B@Gel and X@Gel groups and were markedly suppressed in the XB@Gel group (Figure 7A). Gross liver images and H&E staining confirmed significantly fewer intrahepatic metastatic nodules in the XB@Gel group compared with all other groups (Figure 7B,C). FCM of spleen tissues showed the most prominent increase in CD8^+^ T cells and decrease in Tregs in the XB@Gel group, indicating its excellent role in activating systemic anti‐tumor immunity (Figure 7D).

To further verify the inhibitory effect of XB@Gel on CTCs, blood samples were collected at 1, 3, and 8 days post‐treatment and analyzed by FCM and microscopy. On day 3, GFP^+^ CTC counts in the XB@Gel group were significantly lower than in other groups, with a reduction of more than 54% versus the single‐drug X@Gel group (Figure 7E; Figure S9). IHC concurrently confirmed that VCAM‐1 expression was substantially downregulated in residual cancer tissues of the XB@Gel group (Figure 7F). These results indicate that XB@Gel blocks the CRYAB/β‐catenin pathway to restrain VCAM‐1/β‐integrin‐mediated CTC cluster formation and reduce circulating metastatic seeds, while potentiating systemic immunity to robustly suppress liver metastasis. By achieving one‐target multi‐blockade of phenotypic transition, immune suppression, and CTC‐driven metastasis, XB@Gel represents a precision intervention for post‐iRFA HCC.

## 4 Discussion

This study indicates the critical clinical challenge of iRFA‐accelerated invasion and metastasis of residual HCC. We demonstrate that sublethal thermal stress induces upregulation of the heat‐responsive molecular chaperone CRYAB, which stabilizes β‐catenin and inhibits its ubiquitin‐proteasomal degradation. The CRYAB/β‐catenin axis simultaneously drives EMT in residual cancer cells and polarizes TANs toward the N2 phenotype, thereby pre‐programming a pro‐metastatic propensity in situ and promoting CTC cluster formation, ultimately accelerating residual tumor recurrence and liver metastasis. To disrupt this pathological cascade, we engineered an MMP‐9‐responsive thermosensitive hydrogel (XB@Gel) that enables localized and sustained release of the β‐catenin inhibitor XAV‐939 and the PD‐L1 inhibitor BMS‐202, while simultaneously depleting MMP‐9 in the residual tumor. This strategy reverses EMT and immunosuppressive TME, potently suppressing HCC recurrence and liver metastasis following iRFA. Our findings provide a novel perspective on the mechanisms initiating residual tumor metastasis and offer a translatable therapeutic strategy to improve post‐iRFA outcomes in HCC.

Recent studies have established that iRFA promotes residual tumor progression through various mechanisms including EMT, immune microenvironment remodeling, and epigenetic alterations [7, 8, 10, 37, 38]. However, whether and how these changes are coordinated into a unified thermal stress‐to‐metastasis cascade has remained unclear. Our results indicate that CRYAB acts as a core stress‐responsive molecule that transduces thermal stress into a pro‐metastatic cascade. Knockdown of CRYAB markedly reverses the metastatic capacity of residual cancer cells induced by thermal stress, confirming CRYAB as an upstream regulator of EMT. Moreover, high CRYAB expression is closely associated with poor prognosis in HCC patients, suggesting its potential as a prognostic biomarker following iRFA. Importantly, we further reveal that β‐catenin in the iRFA‐induced TME undergoes a non‐canonical activation pattern independent of classical Wnt pathway alterations, and that its stable activation is dependent on CRYAB. These findings not only provide a mechanistic explanation for our previous observation that thermal stress downregulates E‐cadherin, but also expand the understanding of β‐catenin regulation under stress‐exposed microenvironments.

iRFA is not merely a state of residual tumor but rather a critical stress event driving HCC malignant progression [6, 39]. Sublethal thermal stress dually remodels the oncogenic program of cells and the immunosuppressive TME [40]. This is consistent with our research findings. Specifically, thermal stress induces EMT and enhances migration and invasion of residual cancer cells, while also promoting N2 polarization of TANs and thereby attenuating immune surveillance. Our study implies that mesenchymal‐like tumor cells and N2‐polarized TANs cooperate to form CTC clusters in situ within the residual HCC, a process directly governed by the CRYAB/β‐catenin pathway. This is the first report showing that EMT‐driven residual HCC cells and pro‐tumorigenic immune cells synergistically promote CTC cluster formation through enhanced cell‐cell interactions. It provides a novel mechanistic explanation for the high rates of recurrence and metastasis post‐iRFA and offers insights into the origin of CTC clusters in other tumor types. Given that CTC clusters are rare, short‐lived in circulation, and difficult to target directly [41], we propose that focusing on the residual tumor in situ to block the upstream CRYAB/β‐catenin signaling axis represents a more efficient strategy to prevent post‐iRFA metastasis.

Because residual cancer cells are occult and difficult to identify [42], we propose an intervention strategy that holistically targets the core signaling axis driving residual tumor invasion and metastasis. This approach is better aligned with the clinical scenario of combination therapy following iRFA. As a central component of the canonical Wnt pathway, β‐catenin has long been a hotspot for drug development, with several inhibitors (e.g., XAV‐939) having entered preclinical studies [43]. XAV‐939 promotes β‐catenin ubiquitination and degradation, directly counteracting the stabilizing effect of CRYAB on β‐catenin. Given the dual oncogenic and immunomodulatory functions of β‐catenin, the combination of a β‐catenin inhibitor with an immune checkpoint inhibitor enhances CD8^+^ T cell infiltration and improves anti‐tumor immunity. In contrast, no highly specific CRYAB inhibitor is currently available, and the evolutionary conservation of its chaperone activity poses challenges for therapeutic targeting. Therefore, targeted degradation of β‐catenin, rather than direct inhibition of CRYAB, represents a more clinically translatable therapeutic strategy for residual HCC after iRFA.

To overcome the limitations associated with systemic administration of XAV‐939, we developed an MMP‐9‐responsive thermosensitive hydrogel, XB@Gel, whose components are clinically applicable, offering favorable translational prospects. XB@Gel undergoes in situ gelation to form a local drug depot, reducing systemic exposure and off‐target toxicity. Gelatin, as a specific substrate for MMP‐9 [44, 45], enables triggered drug release specifically within the residual tumor microenvironment while depleting MMP‐9, further reducing metastatic potential. The combination of XAV‐939 and BMS‐202 exerts synergistic effects: XAV‐939 promotes TAN polarization toward the anti‐tumorigenic N1 phenotype and increases CD8^+^ T cell infiltration, whereas BMS‐202 relieves T cell immunosuppression and enhances cytotoxicity. By targeting the CRYAB/β‐catenin axis, XB@Gel simultaneously blocks malignant phenotypic transformation of residual HCC cells, immunosuppression, and CTC cluster formation, achieving a precise, low‐toxicity, and multi‐dimensional anti‐metastatic intervention.

Although CRYAB has been previously reported as a prognostic marker in HCC [46], its role in the context of thermal stress and post‐ablation tumor progression has remained unexplored. Our study establishes a mechanistic framework that directly links thermal stress to metastasis through the CRYAB/β‐catenin axis. Specifically, we identify CRYAB as a thermal sensor activated in residual HCC following iRFA. Heat‐induced CRYAB directly binds to the ARM domain of β‐catenin, inhibiting its ubiquitin‐dependent degradation and thereby stabilizing β‐catenin. This axis synchronously drives EMT and N2‐type TAN polarization, which collectively promote heterotypic CTC–TAN cluster formation. Notably, β‐catenin represents a clinically actionable target, and we developed an MMP‐9‐responsive local hydrogel system, providing a tailored translational intervention for post‐iRFA residual HCC. Collectively, our findings extend the current understanding of CRYAB from a prognostic indicator to a functional driver of post‐ablation metastasis, and offer a rationally designed therapeutic strategy to block this pathological cascade.

We acknowledge the limitations of the filtration‐based CTC enumeration method used in this study. To cross‐validate our CTC enumeration, blood samples from sham and iRFA groups were analyzed using a NextCTC platform. Consistent elevation of CTC counts in the iRFA group was observed with both methods, supporting our conclusion that iRFA promotes CTC shedding. However, this orthogonal validation was restricted to the core comparison (sham vs. iRFA) due to sample limitations. Besides, although the subcutaneous tumor and liver metastasis models used in this study recapitulate fundamental biological features of HCC, they do not fully capture the extensive tumor heterogeneity observed in clinical HCC patients, particularly those with underlying cirrhosis. Our water bath model does not fully mimic clinical RFA. Future work using more relevant thermal profiles (e.g., gradient heating) could further clarify CRYAB/β‐catenin axis activation post‐iRFA.

## 5 Conclusion

This study identifies the CRYAB/β‐catenin axis as the core driver of HCC invasion and metastasis following iRFA. Heat stress‐induced CRYAB inhibits ubiquitin‐mediated degradation of β‐catenin, thereby regulating EMT in residual HCC cells, N2 polarization of TANs, and the formation of heterotypic CTC clusters — the critical initiators of postoperative HCC recurrence and metastasis. We further developed an MMP‐9‐responsive thermosensitive hydrogel for localized and sustained co‐delivery of β‐catenin and PD‐L1 inhibitors to residual tumor sites. This platform effectively blocks the CRYAB/β‐catenin signaling cascade, reverses the malignant EMT of residual HCC cells, remodels an anti‐tumor immune microenvironment, and reduces CTC clusters, ultimately potently inhibiting local recurrence and liver metastasis of HCC after iRFA. This study elucidates a novel molecular mechanism underlying tumor metastasis and provides a highly translatable strategy for precision clinical intervention in HCC following iRFA.

## Author Contributions

**Hui Chen**: Writing – original draft, Conceptualization, Project administration, Investigation, Methodology, Validation, Visualization, Data curation, Software. **Danni Yang**: Writing – review & editing, Project administration, Investigation, Data curation, Visualization, Validation, Software. **Songying Pi**: Writing – review & editing, Software, Methodology, Investigation, Formal analysis. **Yuhan Liu**: Software, Methodology, Investigation, Formal analysis. **Hongbin Huang**: Software, Methodology. **Feile Ye**: Supervision, Methodology. **Yuhong Lin**: Writing – review & editing, Resources, Formal analysis, Supervision. **Zhongzhen Su**: Writing – review & editing, Resources, Project administration, Supervision, Investigation, Formal analysis. **Yongquan Huang**: Writing – review & editing, Conceptualization, Supervision, Project administration, Investigation, Funding acquisition, Formal analysis.

## Ethics approval and consent to participate

All animal experimental procedures were performed under the context of the animal protocol approved by the Laboratory Animal Center of the Fifth Affiliated Hospital of Sun Yat‐sen University (2024060401), and were carried out in compliance with all relevant ethical regulations.

## Conflicts of Interest

The authors declare no conflicts of interest.

## Supporting information




**Supporting File**: advs77578‐sup‐0001‐SuppMat.docx.

## Data Availability

The data that support the findings of this study are available from the corresponding author upon reasonable request.

## References

[1] R. L. Siegel , A. N. Giaquinto , and A. Jemal , “Cancer Statistics, 2024,” CA: A Cancer Journal for Clinicians 74 (2024): 12–49. 38230766 10.3322/caac.21820

[2] G. P. Nagaraju , B. Dariya , P. Kasa , S. Peela , and B. F. El‐Rayes , “Epigenetics in Hepatocellular Carcinoma,” Semin Cancer Biol 86 (2022): 622–632. 34324953 10.1016/j.semcancer.2021.07.017

[3] R. Salem , L. Tselikas , and T. De Baere , “Interventional Treatment of Hepatocellular Carcinoma,” Journal of Hepatology 77, no. 4 (2022): 1205–1206, 10.1016/j.jhep.2022.03.037. 35705428

[4] J. Wu , Z. Zhou , Y. Huang , et al., “Radiofrequency Ablation: Mechanisms and Clinical Applications,” MedComm 5 (2020): 746. 10.1002/mco2.746 PMC11445673 39359691

[5] B. H. Kim , “Surgical Resection versus Ablation for Early Hepatocellular Carcinoma: The Debate Is Still Open,” Clinical and Molecular Hepatology 28, no. 2 (2022): 174–176, 10.3350/cmh.2021.0400. 35078307 PMC9013614

[6] T. Su , M. Huang , J. Liao , et al., “Insufficient Radiofrequency Ablation Promotes Hepatocellular Carcinoma Metastasis through N6‐Methyladenosine mRNA Methylation‐Dependent Mechanism,” Hepatology 74, no. 3 (2021): 1339–1356, 10.1002/hep.31766. 33638162

[7] Z. Xie , L. Wang , Y. Wu , et al., “METTL5 promotes Fatty Acid Metabolism by Modulating Peroxisome to Induce Hepatocellular Carcinoma Recurrence after Thermal Ablation,” Molecular Therapy 34, no. 2 (2026): 1215–1233, 10.1016/j.ymthe.2025.11.016. 41234012 PMC12882658

[8] Z. Xiao , J. Yuan , Q. Wu , et al., “Engineered Bacterial Extracellular Vesicles Mediate Pyroptosis to Counteract m6A Methylation‐based Immunosuppression after Insufficient Radiofrequency Ablation of Hepatocellular Carcinoma,” Acta Pharmaceutica Sinica B 16, no. 1 (2026): 522–538, 10.1016/j.apsb.2025.11.005. 41584339 PMC12827888

[9] X. Liang , Q. Liu , S. Zhu , Z. Li , H. Chen , and Z. Su , “GSDME Has Prognostic and Immunotherapeutic Significance in Residual Hepatocellular Carcinoma after Insufficient Radiofrequency Ablation,” Translational oncology 39 (2024): 101796, 10.1016/j.tranon.2023.101796. 37862939 PMC10589398

[10] X. Chen , Y. Huang , H. Chen , et al., “Augmented EPR Effect Post IRFA to Enhance the Therapeutic Efficacy of Arsenic Loaded ZIF‐8 Nanoparticles on Residual HCC Progression,” Journal of Nanobiotechnology 20, no. 1 (2022): 34, 10.1186/s12951-021-01161-3. 35033089 PMC8760822

[11] Y. Huang , S. Pi , H. Chen , et al., “Ultrasonic‐Controlled Drug Release Prevents Protumorigenic Transition and Improves Sequential Targeting Effect to Enhance Treatment of Residual Hepatocellular Carcinoma,” Biomaterials Research 29 (2025): 0114, 10.34133/bmr.0114. 39882403 PMC11775379

[12] H. Chen , S. Zhang , D. Yang , et al., “Injectable Thermosensitive Hydrogel Targeting STAT3 Reprograms Neutrophils to Amplify Anti‐Tumor Immunity Post‐Radiofrequency Ablation in HCC,” Adv Healthc Mater 15, no. 29 (2026): 71386, 10.1002/adhm.71386. PMC13447948 42363704

[13] X. Huang , E. Nepovimova , V. Adam , et al., “Neutrophils in Cancer Immunotherapy: Friends or Foes?” Molecular Cancer 23, no. 1 (2024): 107, 10.1186/s12943-024-02004-z. 38760815 PMC11102125

[14] C. Yang , C. Liu , C. Xia , and L. Fu , “Clinical Applications of Circulating Tumor Cells in Metastasis and Therapy,” Journal of Hematology & Oncology 18, no. 1 (2025): 80, 10.1186/s13045-025-01733-y. 40846962 PMC12374358

[15] A. Gvozdenovic and N. Aceto , “A Growing Entourage for Heterotypic Circulating Tumor Cell Clusters,” Trends in Cancer 11, no. 12 (2025): 1137–1138, 10.1016/j.trecan.2025.10.006. 41168016

[16] S. Camargo , O. Moskowitz , A. Giladi , et al., “Neutrophils Physically Interact with Tumor Cells to Form a Signaling Niche Promoting Breast Cancer Aggressiveness,” Nature Cancer 6, no. 3 (2025): 540–558, 10.1038/s43018-025-00924-3. 40055573

[17] B. M. Szczerba , F. Castro‐Giner , M. Vetter , et al., “Neutrophils Escort Circulating Tumour Cells to Enable Cell Cycle Progression,” Nature 566, no. 7745 (2019): 553–557, 10.1038/s41586-019-0915-y. 30728496

[18] Y. Chen , J. Bei , M. Liu , et al., “Sublethal Heat Stress‐induced O‐GlcNAcylation Coordinates the Warburg Effect to Promote Hepatocellular Carcinoma Recurrence and Metastasis after Thermal Ablation,” Cancer Letters 518 (2021): 23–34, 10.1016/j.canlet.2021.06.001. 34126196

[19] L. Rice , N. Marzano , D. Cox , B. Skewes , A. M. van Oijen , and H. Ecroyd , “Single‐molecule Observations of human Small Heat Shock Proteins in Complex with Aggregation‐prone Client Proteins,” Biochemical Journal 482, no. 09 (2025): 413–432, 10.1042/BCJ20240473. 40241479 PMC12203938

[20] B. Rashidieh , A. L. Bain , S. M. Tria , et al., “Alpha‐B‐Crystallin Overexpression Is Sufficient to Promote Tumorigenesis and Metastasis in Mice,” Experimental hematology & oncology 12, no. 1 (2023): 4, 10.1186/s40164-022-00365-z. 36624493 PMC9830749

[21] D. Caporossi , A. Parisi , C. Fantini , E. Grazioli , C. Cerulli , and I. Dimauro , “AlphaB‐crystallin and Breast Cancer: Role and Possible Therapeutic Strategies,” Cell Stress and Chaperones 26, no. 1 (2021): 19–28, 10.1007/s12192-020-01175-0. 33111264 PMC7736448

[22] M. Xu , X. Gao , J. Zhao , et al., “UFMylation Suppresses Hepatocellular Carcinoma Metastasis by Inhibiting β‐Catenin–Driven Hybrid EMT and NK Cell Evasion,” Cancer Research 86, no. 13 (2026): 3213–3232, 10.1158/0008-5472.CAN-25-4755. 41996487

[23] M. Guo , Z. Yuan , X. Jin , et al., “Inhibition of ICAM1 Diminishes Stemness and Enhances Antitumor Immunity in Glioblastoma via β‐catenin/PD‐L1 Signaling,” Nature Communications 16, no. 1 (2025): 8642, 10.1038/s41467-025-63796-2. PMC12484827 41028719

[24] X. Wang , Y. Tan , Y. Wang , et al., “Classic Protocadherin PCDH10 Functions as a Tumor Suppressive Scaffold Protein Antagonizing Oncogenic WNT/β‐catenin Signaling in Breast Carcinogenesis,” International Journal of Biological Sciences 22, no. 3 (2026): 1480–1495, 10.7150/ijbs.127857. 41608634 PMC12839071

[25] D. Wang , S. Wu , J. He , et al., “FAT4 overexpression Promotes Antitumor Immunity by Regulating the β‐catenin/STT3/PD‐L1 Axis in Cervical Cancer,” Journal of Experimental & Clinical Cancer Research 42, no. 1 (2023): 222, 10.1186/s13046-023-02758-2. 37658376 PMC10472690

[26] J. M. Llovet , R. Pinyol , M. Yarchoan , et al., “Adjuvant and Neoadjuvant Immunotherapies in Hepatocellular Carcinoma,” Nature Reviews Clinical Oncology 21, no. 4 (2024): 294–311, 10.1038/s41571-024-00868-0. PMC11984461 38424197

[27] R. S. Riley , C. H. June , R. Langer , and M. J. Mitchell , “Delivery Technologies for Cancer Immunotherapy,” Nature Reviews Drug Discovery 18, no. 3 (2019): 175–196, 10.1038/s41573-018-0006-z. 30622344 PMC6410566

[28] S. Zhang , Y. Huang , S. Pi , et al., “Autophagy‐amplifying Nanoparticles Evoke Immunogenic Cell Death Combined with Anti‐PD‐1/PD‐L1 for Residual Tumors Immunotherapy after RFA,” Journal of Nanobiotechnology 21, no. 1 (2023): 360, 10.1186/s12951-023-02067-y. 37789342 PMC10548684

[29] S. Chen , Q. Qiu , D. Wang , et al., “Dual‐sensitive Drug‐loaded Hydrogel System for Local Inhibition of Post‐surgical Glioma Recurrence,” Journal of Controlled Release 349 (2022): 565–579, 10.1016/j.jconrel.2022.07.011. 35835399

[30] A. Chen , J. Zhu , R. Liu , et al., “Injectable Thermo‐sensitive Hydrogel Enhances Anti‐tumor Potency of Engineered Lactococcus Lactis by Activating Dendritic Cells and Effective Memory T Cells,” Bioact Mater 37 (2024): 331–347. 38694762 10.1016/j.bioactmat.2024.03.023 PMC11061616

[31] T. Yoshida , T. Shiraishi , S. Nakata , et al., “Proteasome Inhibitor MG132 Induces Death Receptor 5 through CCAAT/Enhancer‐binding Protein Homologous Protein,” Cancer Research 65, no. 13 (2005): 5662–5667, 10.1158/0008-5472.CAN-05-0693. 15994939

[32] G. Liu , Y. Wang , Y. Pan , et al., “Hypertonicity Induces Mitochondrial Extracellular Vesicles (MEVs) That Activate TNF‐α and β‐catenin Signaling to Promote Adipocyte Dedifferentiation,” Stem Cell Research & Therapy 14, no. 1 (2023): 333, 10.1186/s13287-023-03558-3. 38115136 PMC10731851

[33] D. Stakheev , P. Taborska , Z. Strizova , M. Podrazil , J. Bartunkova , and D. Smrz , “The WNT/β‐catenin Signaling Inhibitor XAV939 Enhances the Elimination of LNCaP and PC‐3 Prostate Cancer Cells by Prostate Cancer Patient Lymphocytes in Vitro,” Scientific Reports 9, no. 1 (2019): 4761, 10.1038/s41598-019-41182-5. 30886380 PMC6423115

[34] J. Park , S. Park , K. A. Hyun , and H. I. Jung , “Microfluidic Recapitulation of Circulating Tumor Cell–Neutrophil Clusters via Double Spiral Channel‐Induced Deterministic Encapsulation,” Lab on a Chip 21, no. 18 (2021): 3483–3497, 10.1039/D1LC00433F. 34309611

[35] N. Pandey , H. Kaur , M. R. Chorawala , et al., “Interactions between Integrin α9β1 and VCAM‐1 Promote Neutrophil Hyperactivation and Mediate Poststroke DVT,” Blood Advances 8, no. 9 (2024): 2104–2117, 10.1182/bloodadvances.2023012282. 38498701 PMC11063402

[36] B. Wang , J. Chen , J. S. Caserto , X. Wang , and M. Ma , “An in Situ Hydrogel‐Mediated Chemo‐Immunometabolic Cancer Therapy,” Nature Communications 13, no. 1 (2022): 3821, 10.1038/s41467-022-31579-8. PMC9250515 35780226

[37] E. Y. Faraoni , B. J. O'Brien , L. N. Strickland , et al., “Radiofrequency Ablation Remodels the Tumor Microenvironment and Promotes Neutrophil‐Mediated Abscopal Immunomodulation in Pancreatic Cancer,” Cancer Immunology Research 11, no. 1 (2023): 4–12, 10.1158/2326-6066.CIR-22-0379. 36367967 PMC9808367

[38] F. Ao , X. Li , Y. Tan , et al., “STING Agonist‐based Hydrogel Enhances Immune Activation in Synergy with Radiofrequency Ablation for Hepatocellular Carcinoma Treatment,” Journal of Controlled Release 369 (2024): 296–308, 10.1016/j.jconrel.2024.01.048. 38301925

[39] Y. Zhou , X. Liu , W. Zhang , et al., “HMGB1 released from Dead Tumor Cells after Insufficient Radiofrequency Ablation Promotes Progression of HCC Residual Tumor via ERK1/2 Pathway,” International Journal of Hyperthermia 40, no. 1 (2023): 2174709, 10.1080/02656736.2023.2174709. 36755436

[40] Y. Zhao , T. Yang , Y. Ouyang , et al., “Radiofrequency Ablation Plays Double Role in Immunosuppression and Activation of PBMCs in Recurrent Hepatocellular Carcinoma,” Frontiers in Immunology 15 (2024): 1339213, 10.3389/fimmu.2024.1339213. 38348038 PMC10859425

[41] Y. Su , M. Leng , Q. Yang , et al., “Targeting Circulating Tumor Cell‒Neutrophil Interactions: Nanoengineered Strategies for Inhibiting Cancer Metastasis,” Journal of Nanobiotechnology 23, no. 1 (2025): 449, 10.1186/s12951-025-03522-8. 40528159 PMC12175327

[42] J. Yang , H. Liang , K. Hu , et al., “The Effects of Several Postoperative Adjuvant Therapies for Hepatocellular Carcinoma Patients with Microvascular Invasion after Curative Resection: A Systematic Review and Meta‐analysis,” Cancer Cell International 21, no. 1 (2021): 92, 10.1186/s12935-021-01790-6. 33549093 PMC7868028

[43] X. Wu , H. Que , Q. Li , and X. Wei , “Wnt/β‐catenin Mediated Signaling Pathways in Cancer: Recent Advances, and Applications in Cancer Therapy,” Molecular Cancer 24, no. 1 (2025): 171, 10.1186/s12943-025-02363-1. 40495229 PMC12150590

[44] M. Dadmehr , M. Mortezaei , and B. Korouzhdehi , “Dual Mode Fluorometric and Colorimetric Detection of Matrix Metalloproteinase MMP‐9 as a Cancer Biomarker Based on AuNPs@Gelatin/AuNCs Nanocomposite,” Biosensors and Bioelectronics 220 (2023): 114889, 10.1016/j.bios.2022.114889. 36368143

[45] W. Zhou , Z. Duan , J. Zhao , R. Fu , C. Zhu , and D. Fan , “Glucose and MMP‐9 Dual‐responsive Hydrogel with Temperature Sensitive Self‐adaptive Shape and Controlled Drug Release Accelerates Diabetic Wound Healing,” Bioact Mater 17 (2022): 1–17. 35386439 10.1016/j.bioactmat.2022.01.004 PMC8958327

[46] Y. Jin , P. Hu , Y. Dai , W. Gu , J. Han , and H. Song , “Nuclear Factor IA‑Mediated Transcriptional Regulation of Crystallin αB Inhibits Hepatocellular Carcinoma Progression,” Molecular and Clinical Oncology 23, no. 2 (2025): 1–14, 10.3892/mco.2025.2867. PMC12210110 40599718

