# Supplementary material for: Targeting the CRYAB/β‐Catenin Axis with an MMP‐9‐Responsive Hydrogel Disrupts CTC Clusters to Suppress Progression of Hepatocellular Carcinoma after Radiofrequency Ablation
Source: Adv Sci (Weinh). 2026 Sep 16:e77578. Online ahead of print. doi: 10.1002/advs.77578 (PMC13583229; doi:10.1002/advs.77578)
Supplement: Supplementary file 1 [file ADVS-9999-e77578-s001.docx]

**Supplementary Figure**

**Figure S1. CRYAB is elevated in residual HCC post-iRFA and drives EMT**
(A) Heat map of RNA‑seq analysis in HCC cells subjected to sublethal heat stress (44 °C) versus control (37 °C). (B) The ranking of the top six upregulated genes. (C) qPCR quantification of CRYAB mRNA in HCC cells under the indicated heat treatments (n=3). (D) Relative luciferase activity (firefly/Renilla) in HCC cells treated at the indicated temperatures (n=3). (E) Transwell migration and invasion assays and quantification in heat‑stressed Hep3B cells with siNC or si*CRYAB* (n=3). Scale bar: 100 µm. (F) Western blot of Vimentin, E‑cadherin, and N‑cadherin in heat‑stressed Hep3B cells with siNC or si*CRYAB* (n=3). (Data are means ± SD. Calculated by Student’s t‑test or One-way ANOVA. **p* < 0.05, ***p* < 0.01, ****p* < 0.001, *****p* < 0.0001).

**Figure S2. qPCR screening of cytokine expression in heat‑stressed HCC cells with CRYAB knockdown.**
(A) qPCR quantification of cytokine mRNAs (G‑CSF, IL‑6, CXCL1, VEGF‑α, TNF‑α, IL‑10, IFN‑β, CXCL12, IL‑17) in heat‑stressed HCC cells with sgNC or sg*Cryab* (n=5). (B) Extended qPCR validation of TGF‑β and MMP‑9 mRNA expression in heat‑stressed HCC cells with si*Cryab*‑a or si*Cryab*‑b (n=5). (Data are means ± SD. Calculated by One-way ANOVA. **p* < 0.05, ***p* < 0.01, ****p* < 0.001, *****p* < 0.0001, ns, not significant).

**Figure S3. CRYAB knockdown suppresses iRFA‑induced residual tumor recurrence and remodels the immune microenvironment.**
(A) Representative gross images of subcutaneous residual tumors from sgNC+sham, sgNC+iRFA, sg*Cryab*+sham, and sg*Cryab*+iRFA groups at day 21 (n=3). (B) Tumor growth curves showing tumor volume over time in the four groups (n=3). (C) Tumor volume at day 21 in the four groups (n=3). (D) IHC staining and H‑score quantification of CRYAB in subcutaneous residual tumors from sgNC+sham, sgNC+iRFA, sgCryab+sham, and sgCryab+iRFA groups (n=3). Scale bar: 200 µm. (E) FCM analysis and quantification of CD8⁺ T cells, TAN, and Tregs (CD4⁺Foxp3⁺) infiltrating residual tumor tissues in the four groups (n=3). (Data are means ± SD. Calculated by One-way ANOVA. **p* < 0.05, ***p* < 0.01, ****p* < 0.001, *****p* < 0.0001).

**Figure S4. CRYAB interacts with β‑catenin.**
(A) Kyoto Encyclopedia of Genes and Genomes (KEGG) enrichment analysis of pathways from the RNA‑seq dataset. (B) STRING protein–protein interaction network analysis identifying β‑catenin (CTNNB1) as a potential CRYAB‑interacting partner. Energy decomposition identified CRYAB residues GLU47, GLU43, and ASP46 (C) and β‑catenin residues LYS181, LYS180, and ARG185 (D) as the major binding contributors. (E) Binding free energy components (van der Waals, electrostatic, polar solvation, and nonpolar solvation) calculated by MMPBSA.

**Figure S5. CRYAB interacts with β‑catenin and regulates downstream signaling.**

(A) qPCR quantification of β‑catenin mRNA in HCC cells treated at 37, 42, 44, and 46 °C (n=3). (B) Western blot of CRYAB and β‑catenin under the same heat treatments (n=3). (C) Western blot of CRYAB and β‑catenin in heat‑stressed HCC cells with or without CRYAB knockdown (siCryab‑a, siCryab‑b) (n=3). (D) CCK-8 assay showing the dose-dependent effects of XAV-939 on HCC cell viability and the determination of its half-maximal inhibitory concentration (IC₅₀) (n=3). (E) Western blot of N‑cadherin and Vimentin in heat‑stressed HCC cells with *Cryab^OE^* and XAV‑939 treatment (n=3). FCM analysis and quantification of iNOS⁺ TANs and CD206⁺ TANs cultured in (F) heated or (G) *Cryab^OE^* TCM and treated with SB-431542 or SB-3CT (n=3). (H) IHC staining and H‑score quantification of β‑catenin in residual tumor tissues from sgNC+sham, sgNC+iRFA, sg*Cryab*+sham, and sg*Cryab*+iRFA groups (n=3). Scale bar: 200 µm. (Data are means ± SD. Calculated by One-way ANOVA. **p* < 0.05, ***p* < 0.01, ****p* < 0.001, *****p* < 0.0001).

**Figure S6.** **iRFA elevates CTCs levels.**

（A）Quantification showing GFP⁺ CTCs (HCC cells) in peripheral blood at 1, 3, and 8 days after iRFA or sham operation (n=3).

**Figure S7. Characterization of XB@Gel.**
(A) Storage modulus (G′) of hydrogels formulated with increasing gelatin concentrations. (B) In vitro degradation profiles of the hydrogels with different gelatin concentrations. (C) Cumulative release profiles of FITC from hydrogels with different gelatin concentrations over 14 days. (D) Viscosity (η) versus shear rate of hydrogels with different gelatin concentrations, illustrating shear-thinning behavior. (E) Encapsulation efficiency of X@Gel and XB@Gel. (F) Photographs demonstrating continuous injection of XB@Gel through a 24G needle into 37 °C PBS. (G) In vivo retention and quantification of XB@Gel in the subcutaneous site at 1, 3, 7, and 14 days post‑injection (n = 3).

**Figure S8. Biosafety evaluation of XB@Gel.**
(A) H&E staining of major organs (heart, liver, spleen, lung, and kidney) harvested from mice 14 days after subcutaneous injection after different treatments. No obvious pathological changes or organ damage were observed across all groups (n=3). Scale bar: 100 μm. (B) Statistical plots showing the liver and renal function, the detection indexes included alanine aminotransferase (ALT), aspartate aminotransferase (AST), total bilirubin (TBIL), urea, creatinine (CRE), and UA in PBS, Gel, XAV, B@Gel, X@Gel, and XB@Gel (n=3).

**Figure S9. XB@Gel reduces CTC counts in peripheral blood after iRFA.**
Representative fluorescence images showing GFP⁺ CTCs (HCC cells) in peripheral blood at 1, 3, and 8 days after iRFA and treatment in the five groups: control, XAV, B@Gel, X@Gel, and XB@Gel (n=3). Scale bar: 30 µm.

**Table S1 Molecular docking**

**Table S2 qPCR primer**

| Gene | Primer | Sequence (5′→3′) |
| --- | --- | --- |
| Mmp9 | Forward | 5′-GGACCCGAAGCGGACATTG-3′ |
|  | Reverse | 5′-CGTCGTCGAAATGGGCATCT-3′ |
| Csf3 | Forward | 5′-GCACTATGGTCAGGACGAGAG-3′ |
|  | Reverse | 5′-GGGGAAATACCCGATAGAGCC-3′ |
| Cxcl1 | Forward | 5′-ACTGCACCCAAACCGAAGTC-3′ |
|  | Reverse | 5′-TGGGGACACCTTTTAGCATCTT-3′ |
| Cxcl15 | Forward | 5′-TCGAGACCATTTACTGCAACAG-3′ |
|  | Reverse | 5′-CATTGCCGGTGGAAATTCCTT-3′ |
| Tgfb1 | Forward | 5′-TGATACGCCTGAGTGGCTGTCT-3′ |
|  | Reverse | 5′-CACAAGAGCAGTGAGCGCTGAA-3′ |
| Ifnb1 | Forward | 5′-AGCTCCAAGAAAGGACGAACA-3′ |
|  | Reverse | 5′-GCCCTGTAGGTGAGGTTGAT-3′ |
| Tnf | Forward | 5′-CCCTCACACTCAGATCATCTTCT-3′ |
|  | Reverse | 5′-GCTACGACGTGGGCTACAG-3′ |
| Cxcl12 | Forward | 5′-TGCATCAGTGACGGTAAACCA-3′ |
|  | Reverse | 5′-CACAGTTTGGAGTGTTGAGGAT-3′ |
| Il10 | Forward | 5′-CGGGAAGACAATAACTGCACCC-3′ |
|  | Reverse | 5′-CGGTTAGCAGTATGTTGTCCAGC-3′ |
| Vegfa | Forward | 5′-GCACATAGAGAGAATGAGCTTCC-3′ |
|  | Reverse | 5′-CTCCGCTCTGAACAAGGCT-3′ |
| Il17a | Forward | 5′-TCAGCGTGTCCAAACACTGAG-3′ |
|  | Reverse | 5′-CGCCAAGGGAGTTAAAGACTT-3′ |
| Cryab | Forward | 5′-CGGAGGAACTCAAAGTCAAGGTT-3′ |
|  | Reverse | 5′-ATGGTGAGAGGATCCACATCGG-3′ |
| Ctnnb1 | Forward | 5′-CCCAGTCCTTCACGCAAGAG-3′ |
|  | Reverse | 5′-CATCTAGCGTCTCAGGGAACA-3′ |
| Vcam1 | Forward | 5′-AGTTGGGGATTCGGTTGTTCT-3′ |
|  | Reverse | 5′-CCCCTCATTCCTTACCACCC-3′ |
